# Effects of gestational protein restriction on autophagy dynamics during odontogenesis

**DOI:** 10.1007/s00441-026-04079-0

**Published:** 2026-06-19

**Authors:** Bruno Calsa, José Antônio Rocha Gontijo, Milton Santamaria-Jr, Patrícia Aline Boer

**Affiliations:** 1 Fetal Programming and Hydroelectrolyte Metabolism Laboratory, Department of Internal Medicine, Faculty of Medical Sciences, Campinas State University (UNICAMP), Rua Cinco de Junho, 350, Cidade Universitária Zeferino Vaz, Campinas, SP 13087-051 Brazil; 2 National Institute of Science and Technology in Developmental Origins of Health and Disease (INCT-DOHaD Brasil), Campinas, Brazil; 3 Department of Social and Pediatric Dentistry, Institute of Science and Technology, College of Dentistry, São Paulo State University (UNESP), São José Dos Campos, SP Brazil

**Keywords:** Undernutrition, Tooth development, Autophagy, DOHaD

## Abstract

Autophagy is a cellular process essential for maintaining homeostasis and supporting development. Herein, we aimed to evaluate the effects of gestational protein restriction (GPR) on tooth germ autophagy. CAG-RFP-EGFP-LC3 female mice were mated and randomly assigned to normal protein or low-protein groups. Embryos were collected, and autophagy flux in the molar tooth germ was quantified. At the bud stage, GPR induced a decrease in autolysosomes in the condensed mesenchyme, alongside an increase in autophagosomes in the dental epithelium. At the cap stage, an increase in lysosome markers was observed in the dental papilla, while a decrease in autolysosomes was observed in enamel organ. By the bell stage, dental papilla exhibited a decrease in autolysosomes and an increase in lysosome markers. Similarly, inner enamel epithelium showed reduced autolysosomes and elevated lysosome markers. Bud stage was the most significantly affected; we further quantified apoptosis and mitosis, revealing impaired cell proliferation and enhanced apoptosis. Our data suggest that GPR block or delay acidification of autophagic vesicles, potentially impairing the recycling of cellular components critical for tooth germ proliferation and differentiation.

**Graphical abstract:**

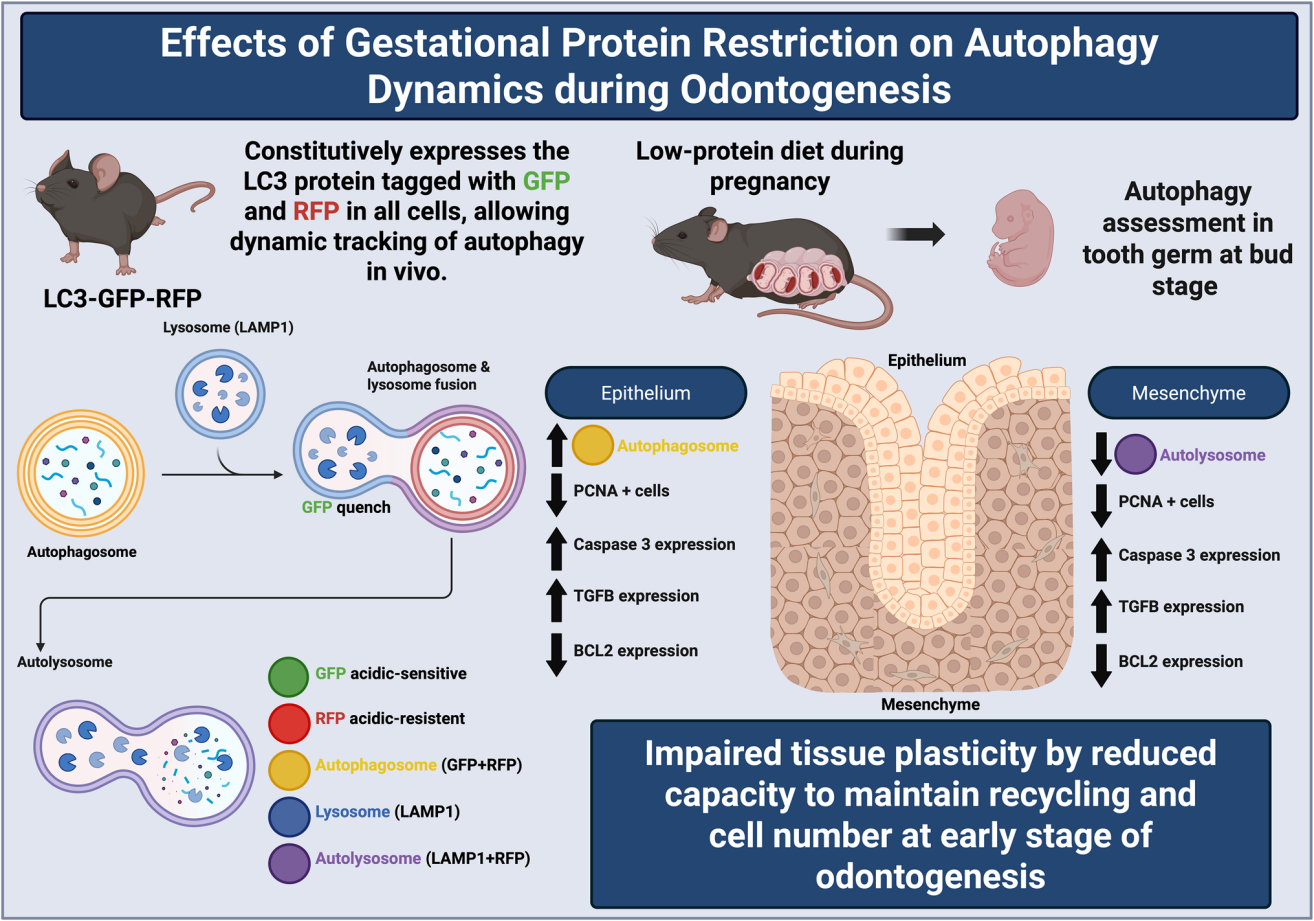

**Supplementary Information:**

The online version contains supplementary material available at 10.1007/s00441-026-04079-0.

## Introduction

Dental development is a highly coordinated process that involves sequential stages, including the bud, cap, and bell stages. These stages reflect the progressive differentiation and morphogenesis of epithelial and mesenchymal tissues that culminate in tooth formation. The bud stage involves cellular proliferation and epithelial-mesenchymal interactions, initiating tooth germ formation. The cap stage is characterized by further differentiation, leading to the establishment of distinct cellular compartments, while the bell stage involves the terminal differentiation of odontoblasts and ameloblasts (Thesleff et al. 1995; Thesleff 2003, 2014; Eldeeb et al. 2024).

These developmental events require tightly coordinated metabolic activity to sustain cellular proliferation, differentiation, protein turnover, and extracellular matrix synthesis. In this context, tooth development is increasingly recognized as a metabolically regulated process in which cellular metabolic status actively interacts with classical developmental signaling pathways, including WNT, BMP, FGF, and Hedgehog signaling (Cao et al. 2026). Therefore, the developing tooth germ may be particularly susceptible to maternal nutritional imbalance during gestation.

Autophagy, a conserved cellular process, is critical for maintaining intracellular homeostasis by degrading and recycling damaged organelles and macromolecules. This process is tightly regulated and involves the formation of autophagosomes, which encapsulate cellular components and fuse with lysosomes for degradation (Glick et al. 2010; Yang et al. 2021).

During organogenesis, cells undergo remodeling processes that require mechanisms for degrading and recycling obsolete cellular components, a function carried out by autophagy (Wang et al. 2019). Disruptions in autophagy have been linked to impaired cellular function and are increasingly recognized as contributors to developmental abnormalities in many organs, including the heart (Lee et al. 2014), kidney (Zhang et al. 2017), and bone (Srinivas and Shapiro 2006; Srinivas et al. 2009; Liu et al. 2013). Similar mechanisms are thought to be important during tooth development, which involves intense tissue remodeling. Microtubule-associated protein 1 light chain 3 (LC3), a key marker of autophagic vesicles, has been identified throughout different stages of odontogenesis, and disruption of autophagy-related pathways has been associated with impaired odontogenic differentiation and enamel defects (Yang et al. 2013b, a; Zhan et al. 2020; Iwaya et al. 2023). More recently, autophagy has also been associated with inflammatory regulation and cellular homeostasis in deciduous dental pulp stem cells (Di et al. 2024a, b). However, its role during embryonic odontogenesis remains poorly understood.

Moreover, conventional static approaches provide limited information regarding autophagic vesicle maturation and flux during tissue development. Recent advances in transgenic models, such as the CAG-RFP-GFP-LC3 mouse (Li et al. 2014), have provided a novel approach to quantify autophagic vesicles through fluorescence-based color changes (Lee et al. 2019). This strain enables the distinction between immature and mature autophagic vesicles—autophagosomes and autolysosomes, respectively, enabling dynamic tracking of autophagic flux in vivo.

Fetal programming refers to the process by which environmental factors during critical periods of development permanently shape the structure, function, and metabolism of tissues and organs (Lucas 1998). This concept was notably advanced by David Barker through the Barker hypothesis, which links maternal nutrition and environmental conditions during pregnancy to long-term health outcomes and disease susceptibility in offspring (Barker et al. 1989a, b; Barker 2004).

Gestational protein restriction (GPR) is a well-established experimental model used to investigate the effects of undernutrition during critical periods of development. Studies of our research group have demonstrated that GPR can disrupt organogenesis, altering cellular processes, leading to long-term physiological consequences in offspring in different organs (Sene et al. 2021b; Lamana et al. 2021; Grigoletti-Lima et al. 2022; Masiero et al. 2022; Folguieri et al. 2022; Calsa et al. 2022, 2024; Gomes et al. 2023; Torres et al. 2024).

This study aimed to investigate the effects of GPR on autophagic dynamics during odontogenesis using transgenic embryos. Our main hypothesis is that GPR disrupts autophagic dynamics during the onset of odontogenesis.

## Materials and methods

### Ethical compliance and experimental design

This study adhered to the Ethical Principles of Animal Experimentation established by the Brazilian College of Animal Experimentation (COBEA), ARRIVE (Animal Research: Reporting of In Vivo Experiments) guidelines, and was approved by the Ethics Committee (Protocol #5923–1/2021 CEUA/UNICAMP). Female transgenic C57BL/6-Tg(CAG-RFP/EGFP/Map1lc3b)1Hill/J mice (RRID: IMSR_JAX:027139), aged 8–10 weeks and weighing 16–18 g, were obtained from the Multidisciplinary Center for Biological Research in Science in Laboratory Animals (CEMIB-UNICAMP). The animals were housed in controlled conditions with a temperature of 23 ± 2 °C, relative humidity of 50 ± 10%, and an inverted light–dark cycle (lights off from 6:00 a.m. to 6:00 p.m.).

To establish the model of gestational protein restriction, 24 transgenic female mice were mated with C57BL/6 J wildtype males during a closely monitored 2-h mating window at the beginning of the dark cycle (8:00 a.m. to 10:00 a.m.). The mating process was directly observed, and the exact time of copulation was recorded. Upon confirmation of pregnancy (gestational day 0, GD0), females were housed individually and allocated into either the normal-protein (NP) group, receiving a diet containing 17% casein, or the low-protein (LP) group, receiving a diet containing 6% casein (Table 1). Both diets were isocaloric and nutritionally balanced for sodium and calcium, as described by Reeves et al. (1993) and our research group (Calsa et al. 2020, 2022, 2024). To ensure developmental stage accuracy, euthanasia and tissue collection were performed at the same hour of the day as the mating event, on the specific gestational days of interest. This timing strategy was adopted to minimize variability due to the rapid and dynamic nature of embryonic development in mice. Pregnant mice were euthanized on GD 14, 16, or 18 using 3% isoflurane anesthesia, and fetuses’ heads were collected for subsequent analyses. To avoid the sexual dimorphic response promoted by fetal programming (Masiero et al. 2022), only male fetuses were used for assessments.

**Table 1:** Composition of standard rodent laboratory diets (AIN 93G): normal-protein (NP) diet, 17% and low-protein (LP) diet, 6%

Component	NP diet (%)	LP diet (%)
Cornstarch	39.67%	48.0%
Dextrinizade starch (90–94%)	13.05%	15.9%
Sucrose	10.0%	12.1%
Carbohydrate	62.72%	76.0%
Casein (84%)	20.23%	7.15%
L-Cysteine	0.3%	0.1%
Cholin bitartrate	0.25%	0.25%
Protein	20.78%	7.5%
Soybean oil	7.0%	7.0%
Total fats	7.0%	7.0%
Cellulose microfine (fiber)	5.0%	5.0%
Fiber	5.0%	5.0%
Mineral mix	3.5%	3.5%
Vitamin mix	1.0%	1.0%
BHT (butylhydroxitoluol)	0.0014%	0.0014%
Energy content	3.80 kcal g^−1^of chow	3.88 kcal g^−1^ of chow

### Sex determination and genotyping

Fetal sex was determined by conventional PCR amplification of the SRY gene as previously described (Calsa et al. 2024). DNA was extracted from tail fragments, and the multiplex PCR reactions of SRY (forward: GTGAGAGGCACAAGTTGGC; reverse: CTCTGTGTAGGATCTTCAATC) and MYG (forward: TTACGTCCATCGTGGACAGC; reverse: TGGGCTGGGTGTTAGTCTTA) genes were performed. Male fetuses were identified by amplification of both SRY (product of 147 pb) and MYG (product of 246 pb), whereas female fetuses showed amplification of MYG only. Genotyping was performed through touchdown PCR for transgene detection following the Jackson Laboratory protocol.

### Immunohistochemical analysis of bud-stage tooth germ

Fetal wildtype heads (*n* = 4/group) at 14 GD were fixed in 4% phosphate-buffered paraformaldehyde, processed for inclusion in Paraplast (Sigma), and coronally sectioned at 4-µm thickness.

For immunoperoxidase, sections were subjected to heat-induced antigen retrieval in citrate buffer (pH 6.0, 0.01 M). Endogenous peroxidase activity was blocked using 0.3% hydrogen peroxide in methanol. To reduce non-specific hydrophobic binding, 3% bovine serum albumin was applied. The following primary antibodies were incubated overnight at 4 °C: BCL-2 (Santa Cruz Biotechnology Cat# sc-492, RRID:AB_2064290, 1:50 dilution), PCNA (Santa Cruz Biotechnology Cat# sc-25280, RRID:AB_628109, 1:1600 dilution), and TGFβ1 (Proteintech Cat# 18,978–1-AP, RRID:AB_11182379, 1:100 dilution). The following secondary antibodies were used: Anti-rabbit IgG, HRP-linked Antibody (Cell Signaling Technology Cat# 7074, RRID: AB_2099233, 1:500 dilution) or Anti-mouse IgG, HRP-linked Antibody (Cell Signaling Technology Cat# 7076, RRID: AB_330924, 1:500 dilution). Finally, the immunostaining was developed using ImmPACT DAB Peroxidase substrate (SK-4105, Vector). The first upper molar germ images were obtained using the photomicroscope BX51 with DP71 camera (Olympus) using UPlanFL 40x/0.75 objective and quantified using ImageJ software (RRID: SCR_003070). Negative control was performed by omitting the primary antibody (Supplementary file 1).

For immunofluorescence, sections were submitted to antigen retrieval as described above. Non-specific bindings were blocked using 5% of non-immune goat serum. The following primary antibodies were incubated overnight at 4 °C: AMPKa1/2 (Santa Cruz Biotechnology Cat# sc-74461, RRID:AB_1118940, 1:200 dilution), Cleaved Caspase-3 Asp175 (Cell Signaling Technology Cat# 9661, RRID:AB_2341188, 1:200 dilution), and mTOR (Sigma-Aldrich Cat# SAB4300583, RRID:AB_10622022, 1:300 dilution). The following secondary antibodies were used: Goat anti-Mouse IgG (H + L) Cross-Adsorbed Secondary Antibody, Alexa Fluor 488 (Thermo Fisher Scientific Cat# A-11001, RRID: AB_2534069, 1:200 dilution), and Cy3 AffiniPure Goat Anti-Rabbit IgG (Jackson ImmunoResearch Labs Cat# 111–165-003, RRID: AB_2338000, 1:200 dilution). The nuclei were stained with DAPI (0.1 µg/mL). The images were obtained using a confocal microscope Upright LSM780-NLO (Carl Zeiss) equipped with laser lines UV 405 nm (for DAPI), Argon 488 nm (for AF 488), and HeNe 561 nm (for Cy3), Plan-Apochromat 40x/1.0 objective with pinholes set to 1 airy unit for each channel and 1024 × 1024 pixels. The first upper molar germ fluorescence intensity was analyzed using ImageJ software. Negative control was performed by omitting the primary antibody (Supplementary file 1).

### Free-floating LAMP1 immunostaining

Fetal transgenic heads (*n* = 4/group/age) were collected and fixed in 4% phosphate-buffered paraformaldehyde as previously described (Calsa et al. 2024). Free-floating coronal cryosections (40 µm) were obtained and blocked with 5% nonimmune donkey serum and subsequently incubated overnight at 4 °C under agitation with a primary antibody against LAMP1 (Novus Cat# NB120-19294, RRID: AB_788858, 1:2000 dilution). Following primary antibody incubation, sections were treated with a secondary antibody conjugated with Alexa Fluor® 647 AffiniPure™ Donkey Anti-Rabbit IgG (Jackson ImmunoResearch Labs Cat# 711–605-152, RRID: AB_2492288, 1:500 dilution).

### Autophagy flux assessment during odontogenesis

We evaluated autophagy dynamics in dental structures by tracking autophagic flux at key gestational stages: 14, 16, and 18 days, corresponding to the bud, cap, and early bell stages, respectively. Regions of interest (ROIs) were selected based on the specific locations of cell lineages responsible for the differentiation into matrix-secreting dental cells, such as odontoblasts and ameloblasts (Jing et al. 2022).

At the bud stage, the dental mesenchyme and dental epithelium were selected as ROIs, as these structures differentiate into odontoblasts and ameloblasts, respectively. For the cap stage, the dental papilla and middle dental epithelium were analyzed. At the early bell stage, the coronal and middle dental papilla were selected to avoid mistakenly including the apical follicle in the analysis, while the inner enamel epithelium was chosen for its critical role in enamel formation.

Images of the whole first upper molar germ were performed using a confocal microscope LSM880 Airyscan (Carl Zeiss) equipped with laser lines: Argon 488 nm (for GFP–green channel), HeNe 543 nm (for RFP–red channel), and HeNe 633 nm (for AF 647–blue channel). The images were captured in tile scan using super-resolution Airyscan mode.

For 14 gestational day images, a C Plan-Apochromat 63x/1.4 Oil DIC objective was used; the resolution of a single image with × 1.8 zoom images was 2124 × 2124 pixels (corresponding to an area of 75.0 × 75.0 microns). For 16 and 18 GD images, an EC Plan-Neofluar 40x/1.3 Oil DIC objective was used; the resolution of a single image with × 1.8 zoom images was 2552 × 2552 pixels (corresponding to an area of 117.0 × 117.0 microns).

Vesicle counting was performed as previously described (Calsa et al. 2024). Vesicles were automatically segmented using a macro in ImageJ software. The macro included the following steps: conversion to 8-bit, application of a Gaussian blur (sigma = 2), subtraction of background with a rolling value of 30, auto-thresholding using the IsoData method, conversion to a mask, application of the watershed algorithm, and removal of particles smaller than 40 pixels. The fluorescence intensity of each vesicle was measured across the red, green, and blue channels, and vesicle colors were classified based on HUE angle ranges: yellow (52–80), orange (9–51), red (0–8, 357–360), purple (263–356), and blue (190–262) using the formula described by Lee et al. (2019). The total number of autophagic vesicles in different tooth germ structures was normalized by area, expressed as the number of vesicles per mm^2^.

### Statistical analysis

Statistical analyses were performed using GraphPad Prism (RRID: SCR_002798), with a significance level set at *p* ≤ 0.05. Data are presented as mean ± standard deviation (SD). Normality of the data distribution was assessed using the Shapiro–Wilk test. For datasets following a normal distribution, comparisons were made using the Student *t*-test. For non-normally distributed data, the Mann–Whitney *U* test was used. Sample sizes and corresponding *p*-values are provided in the figures.

## Results

### Gestational protein restriction on fetal development

The male fetuses’ developmental parameters, including body mass and placental mass, were assessed at 14, 16, and 18 gestational days (GD) in normal protein (NP) and low-protein (LP) groups and are shown in Table 2.

**Table 2:** Fetuses and dams parameters at 14, 16, and 18 gestational day (GD). Results were expressed by mean ± SD and compared using Student’s *t*-test

Parameters (g)	NP	LP	*p* value
Fetuses’ parameters			
Body mass 14 GD	0.19 ± 0.023	0.21 ± 0.02	**0.0103**
Placental mass 14 GD	0.117 ± 0.02	0.118 ± 0.02	0.8985
Body mass 16 GD	0.464 ± 0.03	0.453 ± 0.04	0.4878
Placental mass 16 GD	0.131 ± 0.01	0.110 ± 0.01	**0.0035**
Body mass 18 GD	0.965 ± 0.09	0.931 ± 0.08	0.2730
Placental mass 18 GD	0.137 ± 0.02	0.115 ± 0.02	**0.0083**
Dams’ parameters			
Weight gain 14 GD	9.51 ± 0.8	7.01 ± 0.6	**0.0028**
Weight gain 16 GD	10.74 ± 0.5	7.59 ± 1.6	**0.01**
Weight gain 18 GD	15.9 ± 1.9	10.8 ± 1.7	**0.0073**
Calories consumption 14 GD	188.4 ± 16.7	186.7 ± 16.9	0.8904
Calories consumption 16 GD	207.9 ± 5.7	227.4 ± 12.2	**0.0273**
Calories consumption 18 GD	216.9 ± 30.6	255.1 ± 14.2	0.0643

At 14 GD, the body mass in the LP group was significantly increased compared to the NP group. However, no significant differences were observed for placental mass between groups. By 16 GD, the LP group exhibited a significant reduction in placental mass, suggesting compromised nutrient transport to the fetus. While body mass remained equal between groups. At 18 GD, the reduction in placental mass persisted in the LP group, while body mass showed no significant difference.

The dam’s parameters were also assessed at 14, 16, and 18 GD and are shown in Table 2. In all experimental times, the LP dams showed lower weight gain compared to the NP group. In addition, only in 16 GD, LP dams showed an elevated calorie consumption compared to the NP group.

### GPR accumulates immature autophagic vesicles in bud-stage mesenchyme

**Fig. 1:**
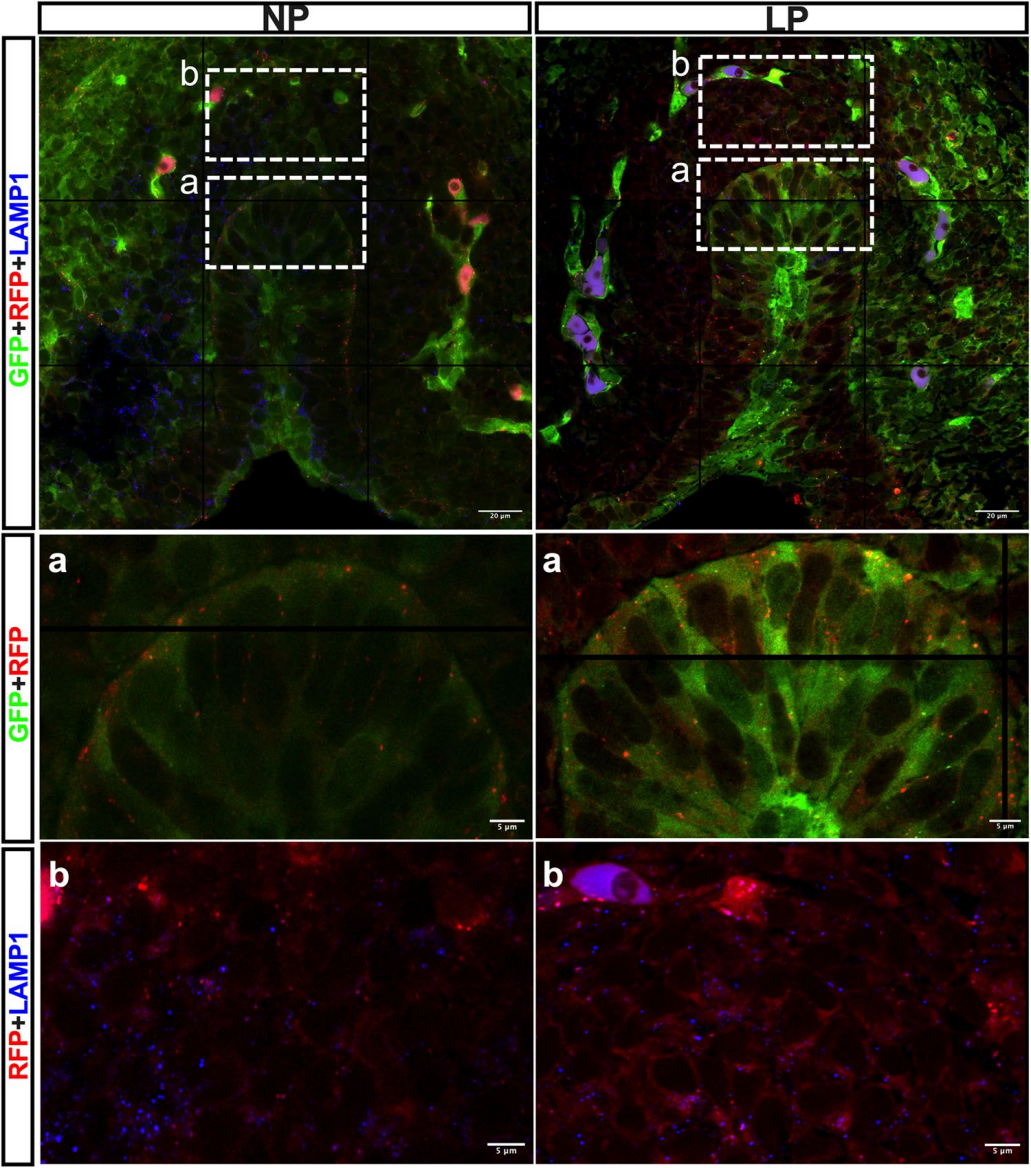
Photomicrograph of autophagy assessment in first molar tooth germ at bud stage. A tile scan composed of 3 × 3 images was acquired using a × 63 objective to capture the whole tooth germ with super-resolution. High-magnification fields show dental epithelium (**a**) and dental mesenchyme (**b**). Scale bars: 20 microns (whole tooth germ) and 5 microns (high-magnification). Note that in epithelium (**a**), a marked increase in autophagosomes (GFP + RFP vesicles) is observed, whereas in the mesenchyme (**b**), an increase in autolysosomes lacking LAMP1 is noted

**Fig. 2:**
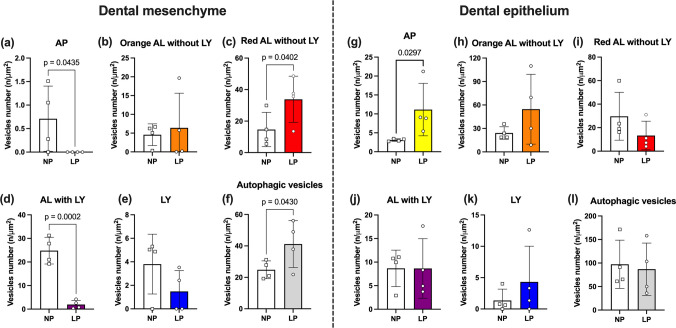
Assessment of autophagy dynamic on tooth germ at bud stage. Dental mesenchyme flux in **a** autophagosomes, **b** orange autolysosomes without lysosome, **c** red autolysosomes without lysosome, **d** autolysosome with lysosome fusion, **e** lysosomes stained with LAMP1, and **f** number of autophagic vesicles. Dental epithelium flux in **g** autophagosomes, **h** orange autolysosomes without lysosome, **i** red autolysosomes without lysosome, **j** autolysosome with lysosome fusion, **k** lysosomes stained with LAMP1, and **l** number of autophagic vesicles. The results were expressed by mean ± SD and compared using the Student *t*-test

We assessed the dynamics of autophagy in the dental mesenchyme and dental epithelium at the bud stage. The representative panels are shown in Fig. 1 and Supplementary File 2. In the dental mesenchyme, we observed a reduction in the number of autophagosomes (Figs. 1a and 2a), an increase in red autolysosomes (Figs. 1a and 2c), a decrease in purple autolysosomes (Figs. 1a and 2d), and an increase in the number of autophagic vesicles (Figs. 1a and 2f). No statistic change was observed in orange autolysosomes (Fig. 2b) and lysosomes (Fig. 2e). In the dental epithelium, we found only an increase in the number of autophagosomes (Figs. 1b and 2g). These results suggest autophagic acidification impairment, which may impair further tooth homeostasis. No statistical change was observed in orange autolysosomes (Fig. 2h), red autolysosomes (Fig. 2i), autolysosomes with LAMP1 (Fig. 2j), lysosomes (Fig. 2k), and autophagic vesicles (Fig. 2l).

### GPR increases lysosomes in the cap-stage dental papilla

**Fig. 3:**
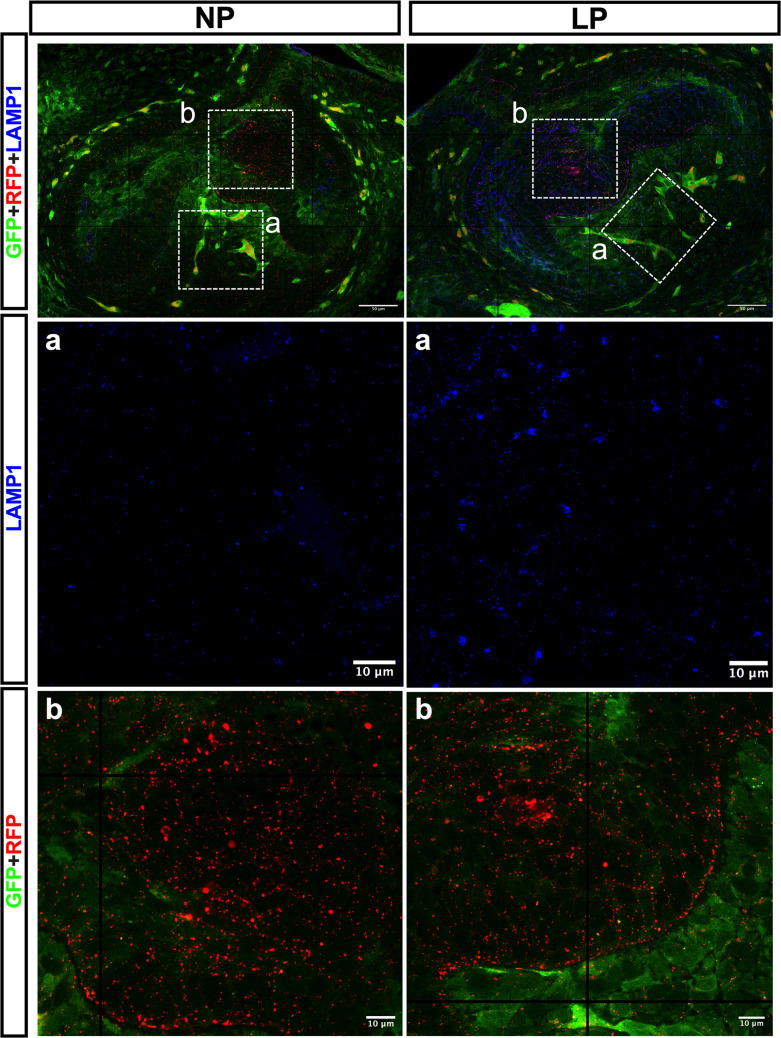
Photomicrograph of autophagy assessment in first molar tooth germ at cap stage. A tile scan composed of 4 × 3 images was acquired using a × 40 objective to capture the whole tooth germ with super-resolution. High-magnification fields show dental papilla (**a**) and enamel organ (**b**). Scale bars: 50 microns (whole tooth germ) and 10 microns (high-magnification). In papilla (**a**), an increase in LAMP1 staining is observed, whereas in the enamel organ (**b**), a decrease in orange autolysosomes is noted

**Fig. 4:**
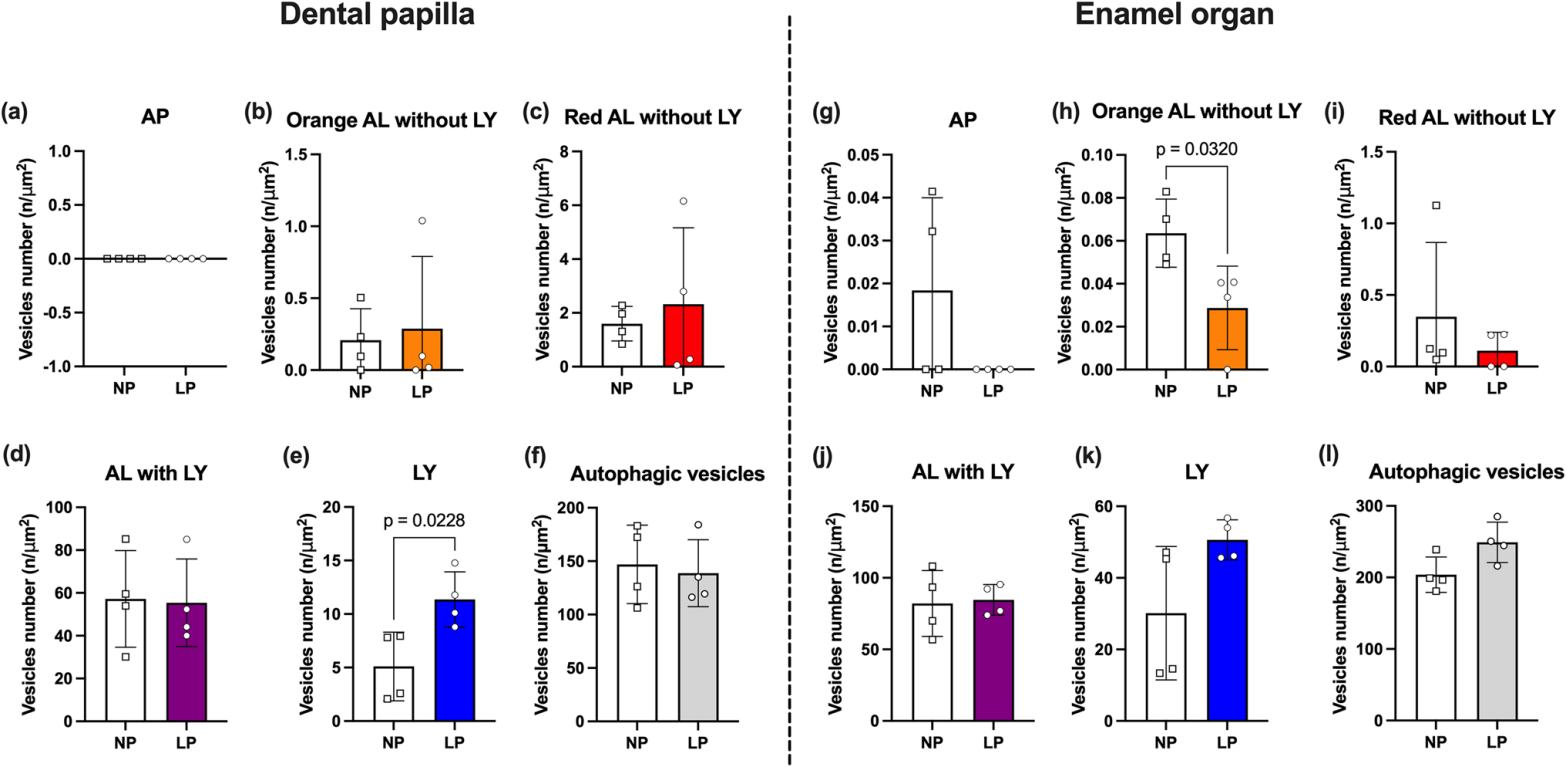
Assessment of autophagy dynamic on tooth germ at cap stage. Dental papilla flux in **a** autophagosomes, **b** orange autolysosomes without lysosome, **c** red autolysosomes without lysosome, **d** autolysosome with lysosome fusion, **e** lysosomes stained with LAMP1, and **f** number of autophagic vesicles. Enamel organ flux in **g** autophagosomes, **h** orange autolysosomes without lysosome, **i** red autolysosomes without lysosome, **j** autolysosome with lysosome fusion, **k** lysosomes stained with LAMP1, and **l** number of autophagic vesicles. The results were expressed by mean ± SD and compared using the Student *t*-test

Next, we assessed the dynamics of autophagy in the dental papilla and enamel organ at the cap stage. The representative panels are shown in Fig. 3 and Supplementary File 3. In dental papilla, we observed an increase in the number of LAMP1 lysosomes (Figs. 3a and 4e). No statistical differences in autophagosomes (Fig. 4a), orange autolysosomes (Fig. 4b), red autolysosomes (Fig. 4c), autolysosomes with LAMP1 (Fig. 4d), and autophagic vesicles (Fig. 4f) were observed. In enamel organ, we found a reduction in orange autolysosomes (Figs. 3b and 4h). No statistical change was found in autophagosomes (Fig. 4g), red autolysosomes (Fig. 4i), autolysosomes with LAMP1 (Fig. 4j), lysosomes (Fig. 4k), and autophagic vesicles (Fig. 4l). These results suggest enhanced lysosome biogenesis in the dental papilla, which may contribute to the restoration or compensation of autophagic activity during tooth development under GPR.

### GPR increases lysosomes in the bud stage in the dental papilla and the inner epithelium

**Fig. 5:**
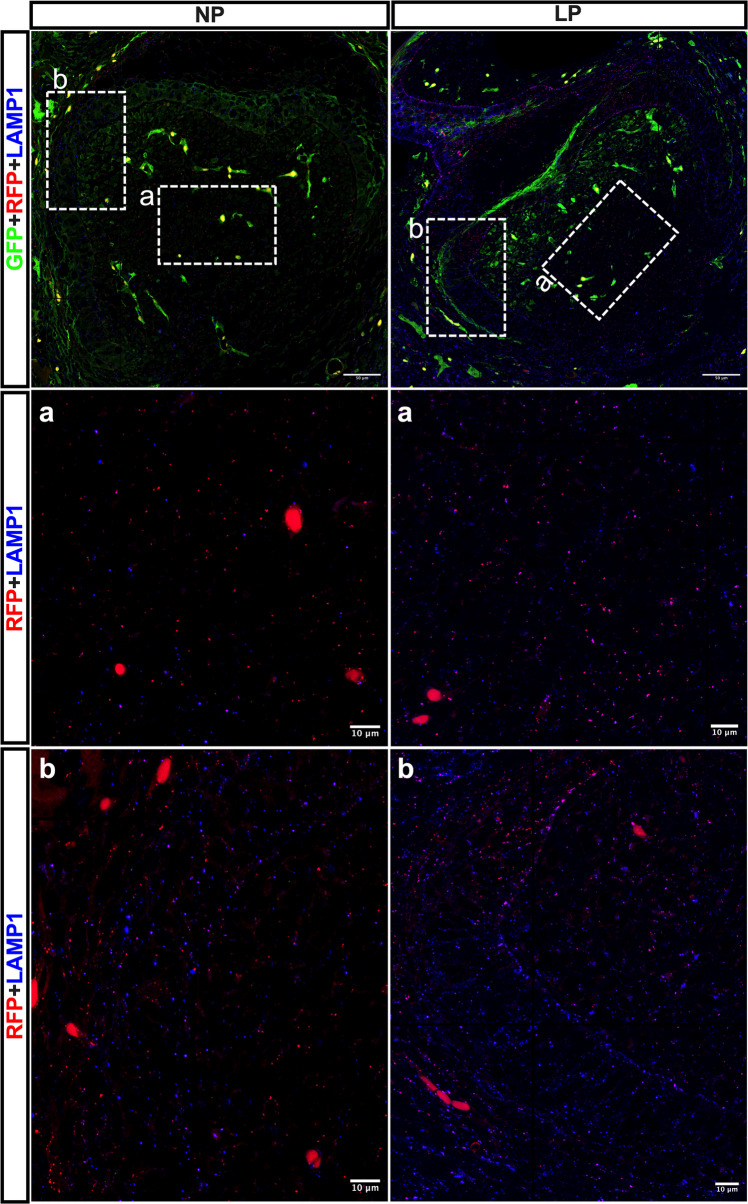
Photomicrograph of autophagy assessment in first molar tooth germ at early bell stage. A tile scan composed of 4 × 4 images was acquired using a × 40 objective to capture the whole tooth germ with super-resolution. High-magnification fields show dental papilla (**a**) and inner enamel epithelium (**b**). Scale bars: 50 microns (whole tooth germ) and 10 microns (high-magnification). In papilla (**a**) and inner enamel epithelium (**b**), an increase in LAMP1 staining is noted

**Fig. 6:**
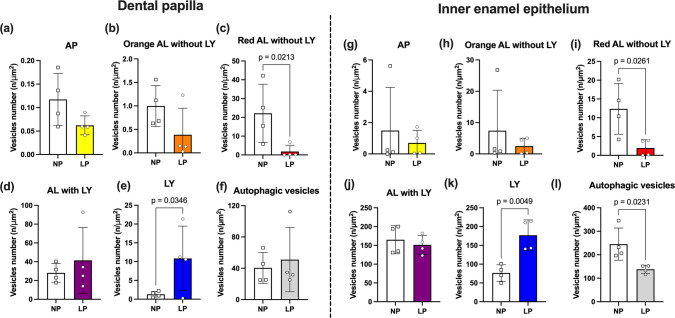
Assessment of autophagy dynamic on tooth germ at early bell stage. Dental papilla flux in **a** autophagosomes, **b** orange autolysosomes without lysosome, **c** red autolysosomes without lysosome, **d** autolysosome with lysosome fusion, **e** lysosomes stained with LAMP1, and **f** number of autophagic vesicles. Inner enamel epithelium flux in **g** autophagosomes, **h** orange autolysosomes without lysosome, **i** red autolysosomes without lysosome, **j** autolysosome with lysosome fusion, **k** lysosomes stained with LAMP1, and **l** number of autophagic vesicles. The results were expressed by mean ± SD and compared using the Student *t*-test

Finally, we assessed the autophagy dynamics in the dental papilla and inner enamel epithelium at the early bell stage. The representative panels are shown in Fig. 5 and Supplementary File 4. In dental papilla, we observed a reduction in red autolysosomes (Figs. 5a and 6c) and an increase in LAMP1 lysosomes (Figs. 5a and 6e). No statistical change in autophagosomes (Fig. 6a), orange autolysosomes (Fig. 6b), autolysosomes with LAMP1 (Fig. 6d), and autophagic vesicles (Fig. 6f) was found. In inner enamel epithelium, we also found a reduction in red autolysosomes (Figs. 5b and 6i) and an increase in LAMP1 lysosomes (Figs. 5b and 6k), in addition to a reduction of autophagic vesicles (Figs. 5b and 6l). No statistical change in autophagosomes (Fig. 6g), orange autolysosomes (Fig. 6h), and autolysosomes with LAMP1 (Fig. 6j) was found. These findings indicate that GPR induces lysosomal accumulation while impairing autophagic flux.

### GPR promotes differentiation-proliferation unbalance in the bud tooth germ

**Fig. 7:**
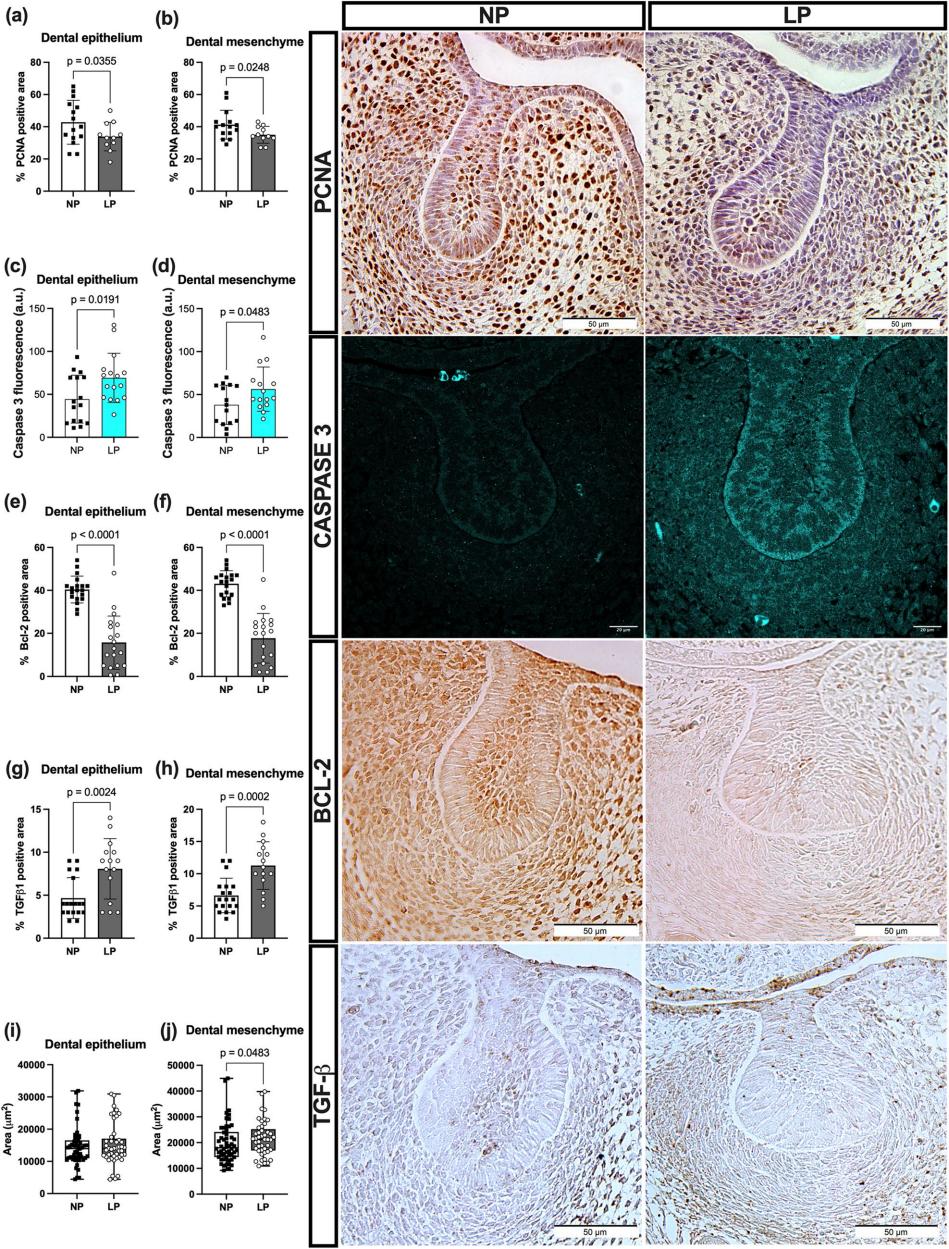
Immunohistochemical assessment of key proteins involved in odontogenesis. Panels (**a**) and (**b**) show the percentage of PCNA-positive area in the dental epithelium and dental mesenchyme, respectively. Panels (**c**) and (**d**) show Caspase-3 fluorescence in the dental epithelium and dental mesenchyme, respectively. Panels (**e**) and (**f**) show the percentage of BCL-2-positive area in the dental epithelium and dental mesenchyme, respectively. Panels (**g**) and (**h**) show the percentage of TGF-β1-positive area in the dental epithelium and dental mesenchyme, respectively. Panels (**i**) and (**j**) show representative images of the dental epithelium and dental mesenchyme, respectively. Representative immunostaining images are shown on the right. Data are presented as mean ± SD and were compared using Student's t-test or the Mann–Whitney test, as appropriate. Scale bar: 20 µm for Caspase-3 and 50 µm for the other proteins

To explore the mechanisms underlying the early odontogenesis, we analyzed the expression of key regulatory proteins that could be influenced by maternal protein restriction, as shown in Fig. 7. At the bud stage, the maternal low-protein diet distinctly modulated the expression of these regulators in the dental epithelium and mesenchyme, which are critical for cell proliferation, apoptosis, and tissue development.

PCNA expression (Fig. 7a and b) was significantly reduced in both the dental epithelium and mesenchyme of LP fetuses, indicating decreased cell proliferation. Conversely, the marker of apoptosis—cleaved Caspase-3 (Fig. 7c and d), showed an increased expression in both tooth germ structures.

The expression of the apoptosis regulator BCL-2 (Fig. 7e and f) was notably reduced in both the dental mesenchyme and epithelium of LP fetuses, further suggesting an enhanced apoptotic environment. Additionally, TGFβ1 expression (Fig. 7g and h) was elevated in LP animals. These results demonstrate that GPR disrupts the balance between cell proliferation and differentiation in the bud-stage tooth germ and alters the expression of key regulatory proteins critical for early tooth development.

Moreover, the area occupied by dental epithelium and condensed mesenchyme was quantified in immunoperoxidase images. No change was found in dental epithelium area (Fig. 7i); however, an increase was found in dental mesenchyme area (Fig. 7j). The immunostaining representative images were shown in the right panel (Fig. 7). Further studies employing high-resolution imaging and single-cell approaches are warranted to clarify the observed mesenchymal expansion and to characterize the specific cell populations within the tooth mesenchyme under GPR.

### GPR increases mTOR expression in the bud-stage dental germ

**Fig. 8:**
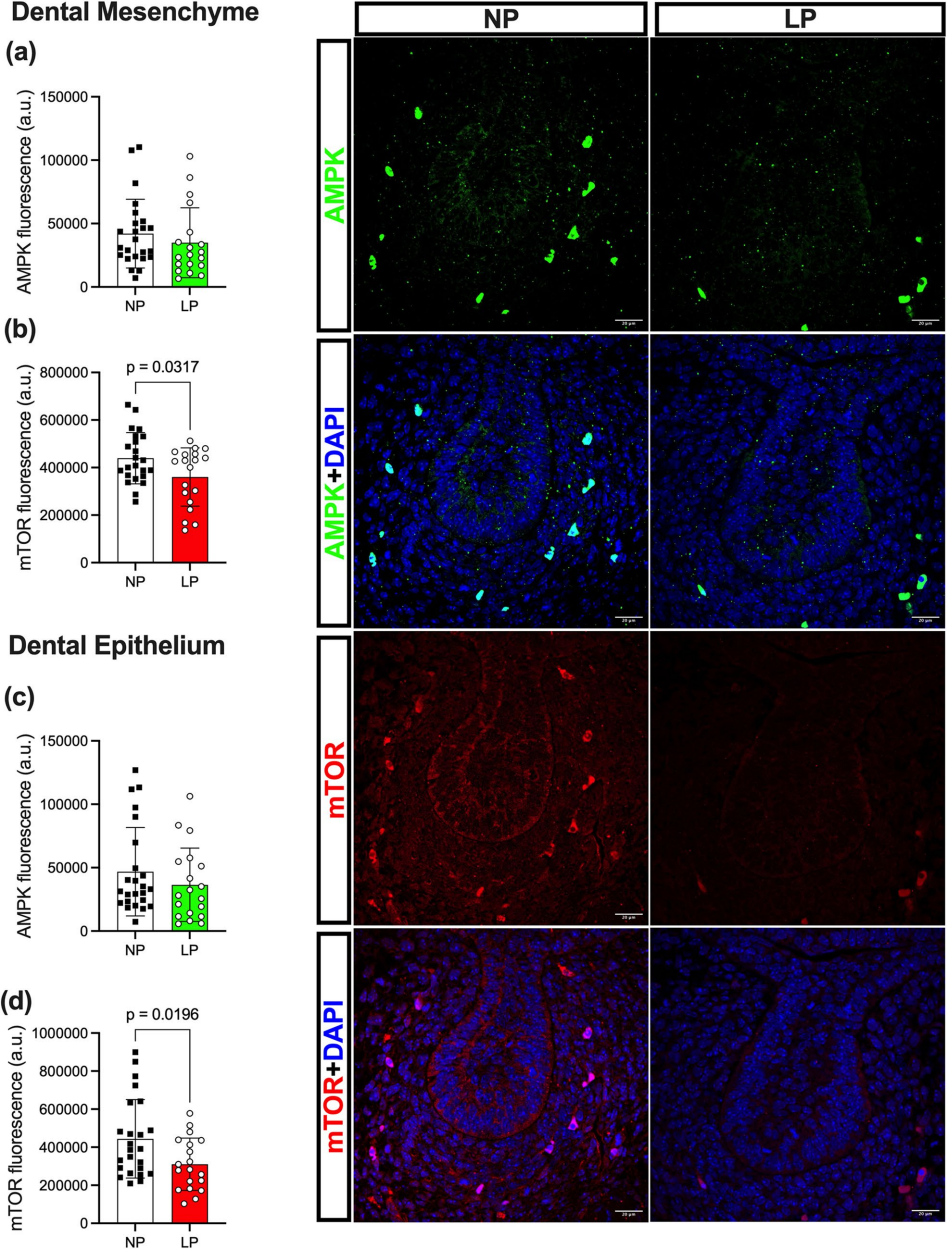
Immunofluorescence assessment of AMPK and mTOR expression during odontogenesis. Panels (**a**) and (**b**) show AMPK and mTOR fluorescence intensity in the dental mesenchyme, respectively. Panels (**c**) and (**d**) show AMPK and mTOR fluorescence intensity in the dental epithelium, respectively. Representative images are shown on the right. Data are presented as mean ± SD and were compared using Student's t-test. Scale bar: 20 µm

To investigate the effects of maternal protein restriction on AMPK and mTOR signaling pathways during tooth development, immunofluorescence staining was performed on dental mesenchyme and epithelium. The results were presented in Fig. 8. Maternal protein restriction reduced mTOR expression in both dental mesenchyme (Fig. 8b) and dental epithelium (Fig. 8d), as evidenced by decreased fluorescence intensity in the LP group. In contrast, AMPK expression remained unchanged in both tissues (Fig. 8a and c). These findings indicate that GPR specifically downregulates mTOR signaling in the bud-stage dental germ without affecting AMPK expression.

## Discussion

Gestational protein restriction (GPR) has been extensively studied for its impact on fetal development, with particular attention to its effects on placental function, fetal, and organ growth. Our findings demonstrate that at 16 and 18 GD, placental mass was significantly lower in LP males. This result aligns with previous studies using a murine model, which have shown that gestational low-protein diets or reduced energy intake induce changes in placental weight and morphology (Rutland et al. 2007; Strakovsky et al. 2010; Coan et al. 2011). Despite the reduction in placental mass, body mass remained unchanged. These effects vary depending on the model, as gestational protein restriction has been shown to cause a significant reduction in placental and/or fetal mass either as early as mid-gestation or only at the end of gestation (Fernandez-Twinn et al. 2003; Jansson et al. 2006; Rutland et al. 2007; Vaughan et al. 2012).

Gonzalez et al. (2016) demonstrated that at the beginning of pregnancy, maternal protein restriction alters the function and growth of the placental junctional zone, and this region remains reduced until the end of pregnancy without any change in fetal weight. The authors suggest that this zone is a sensor of the female’s nutritional status and induces adaptations that mitigate the reduction in fetal growth.

The impact of GPR on tooth development requires a thorough evaluation of its effects during key stages of odontogenesis. Our findings demonstrate that maternal protein restriction disrupts autophagic flux during odontogenesis in a stage- and tissue-specific manner. At the bud stage, we observed an accumulation of immature autophagic vesicles in the dental mesenchyme and elevated autophagosomes in the epithelium, suggesting a blockage in autophagic acidification. These disruptions are associated with reduced cell proliferation and increased apoptosis, indicating impaired cellular homeostasis. Although lysosomal activity appeared to recover at the cap and early bell stages, we speculate that it may be insufficient to fully restore normal tooth germ development.

The bud stage represents a critical phase in odontogenesis, marked by the initiation of epithelial-mesenchymal interactions that drive early tooth morphogenesis. During this period, coordinated cellular proliferation, migration, and condensation of the dental mesenchyme and epithelium establish the foundation for subsequent differentiation of ameloblasts and odontoblasts (Thesleff et al. 1995; Thesleff 2003). Given the central role of cellular processes such as autophagy, cell proliferation, and differentiation in determining cell fate, we hypothesize that their disruption at the bud stage may trigger cascading effects throughout subsequent stages of development, ultimately compromising the structural and functional integrity of the tooth germ.

Previous research by our group demonstrated that at the bell stage (21GD), rats subjected to GPR exhibited reduced dentin thickness and upregulation of the RANKL/OPG pathway (Calsa et al. 2020). Furthermore, at postnatal day 15, these rats displayed an increased RANKL/OPG ratio, reduced vascularity in the dental pulp, and an imbalance in dentin-related gene expression, collectively indicating delayed tooth development (Calsa et al. 2022).

To test our hypothesis that GPR disrupts autophagic dynamics during the onset of odontogenesis, we used a transgenic mouse expressing LC3 protein tagged with two fluorescent markers: GFP (acid-sensitive) and RFP (acid-insensitive). This dual-labeling enables differentiation between autophagosomes and autolysosomes. In the acidic environment of autolysosomes (pH 4–5), the GFP signal is quenched while the RFP signal remains stable. Additional immunostaining for LAMP1 was employed to identify autolysosomes, which express LAMP1 (purple vesicles), and differentiate them from early autolysosomes that lack LAMP1 (red vesicles) (Li et al. 2014).

The importance of autophagy in mineralized tissues has been increasingly recognized (Moran et al. 2025). Preliminary findings from Yang et al. (2013b) demonstrated that LC3 is expressed throughout all stages of odontogenesis; however, its precise roles remain unclear due to the limitations of static immunostaining techniques. In contrast, our study utilized LC3 transgenic mice expressing GFP and RFP reporters, which allow dynamic tracking of autophagic vesicle maturation, distinguishing between autophagosomes and autolysosomes.

The study by Cho et al. (2021) showed that autophagy is upregulated in a tooth cavity model, with increased expression of LC3. Similarly, a genetic model involving Atg7 knockout in epithelial-derived tissues highlighted the essential role of autophagy in ameloblast differentiation. The loss of Atg7 led to disrupted ameloblast function and the development of amelogenesis imperfecta (Iwaya et al. 2023).

In vitro evidence using RNA-seq further indicates that autophagy-related genes are upregulated in dental pulp stem cells during odontogenic differentiation (Chen et al. 2024). Inhibition of autophagy disrupts lysosomal fusion and acidification, reducing odontoblastic differentiation capacity (Zhan et al. 2020). Additionally, autophagy has been observed to play a protective role during odontoblast differentiation under inflammatory conditions, helping maintain cellular function (Pei et al. 2016). These findings suggest that autophagy plays a pivotal role in tooth development, and any impairment in this process could delay or disrupt odontogenesis.

We observed dynamic changes in autophagy during tooth development, particularly at the bud stage. In the dental mesenchyme, there was an increase in red vesicles (RFP + only), suggesting altered vesicle acidification dynamics, accompanied by a reduction in fully acidified autolysosomes (LAMP1 + and RFP +). In the dental epithelium, we noted an increase in yellow puncta (GFP + RFP +), indicative of immature autophagosomes. In mammals, autophagosomes can either fuse directly with lysosomes or first merge with endosomes before progressing to lysosomes, ultimately forming autolysosomes (Berg et al. 1998). During this process, late endosomes exhibit an increasingly acidic environment (Wang et al. 2017).

We hypothesize that, in the dental mesenchyme, early fusion with endosomes may promote vesicle acidification, potentially quenching GFP fluorescence and resulting in a predominant RFP signal. Therefore, the increased number of red vesicles observed in our analysis may reflect enhanced endosomal acidification rather than complete autolysosome formation. Furthermore, as LAMP1 and 2 account for approximately 50% of the lysosomal membrane protein content (Barral et al. 2022), their detection may not fully capture the extent of autophagic vesicle acidification. To support this hypothesis, further experiments are needed, including assessments of lysosomal pH, cathepsin activity, V-ATPase expression, and endosomal markers, to better characterize the maturation and acidification stages of autophagy during odontogenesis.

mTOR is a key negative regulator of autophagy, suppressing ULK1 complex activity (Dunlop and Tee 2014). We previously reported that GPR reduces *mTOR* gene expression in mandible, potentially leading to increased autophagy initiation (Calsa et al. 2024). In parallel, AMPK acts as a central metabolic sensor that promotes autophagy through ULK1 activation and recruitment of ATG proteins under conditions of energy stress (Wang et al. 2022). Together, the AMPK/mTOR axis integrates nutrient and energy availability with developmental signaling pathways involved in odontogenesis, including WNT and BMP signaling, thereby regulating cellular proliferation, differentiation, and metabolic homeostasis during tooth development (Choo et al. 2006; Inoki et al. 2006; Hardie et al. 2012; Rahman et al. 2015; Cao et al. 2026).

Our current findings demonstrate a decrease in mTOR staining, accompanied by an accumulation of autophagosomes in the dental epithelium and a reduction in autolysosomes in the dental mesenchyme. This pattern suggests that while autophagy initiation is not impaired, the maturation and degradation phases may be compromised. Despite the central role of AMPK in autophagy regulation, its expression remained unchanged between groups, suggesting that the observed alterations in autophagic dynamics are predominantly associated with mTOR-dependent pathways. Therefore, reduced mTOR activity induced by GPR may contribute not only to altered autophagic flux, but also to disturbances in developmental signaling and cellular homeostasis during odontogenesis.

One of the fetal programming mechanisms is the excessive exposure to maternal glucocorticoids due to an imbalance of placental enzyme 11β-hydroxysteroid dehydrogenase (Hales and Barker 2001). This imbalance accelerates the differentiation process while impairing cell proliferation, as previously evidenced by our group in other tissues (Sene et al. 2021a). In this context, the observed increase in TGFβ1, a regulator of dental differentiation, and the decrease in PCNA, a marker of cell proliferation, support our hypothesis that GPR disrupts the balance between cellular proliferation and differentiation.

Dental epithelium and mesenchyme disruption during critical stages of odontogenesis may have long-term consequences for oral health. Perturbations in early tooth germ homeostasis and epithelial-mesenchymal interactions may compromise enamel and dentin formation, potentially predisposing offspring to enamel hypoplasia, structural dentin defects, delayed tooth eruption, and increased susceptibility to pulpal and periodontal alterations, as previously suggested (Calsa et al. 2020, 2022, 2024). Altered autophagic activity has also been associated with inflammatory regulation and cellular homeostasis in deciduous dental pulp stem cells (Di et al. 2024a, b), reinforcing the importance of balanced autophagic dynamics for proper tissue development and maintenance. These findings reinforce the concept that adverse intrauterine nutritional environments may influence oral health trajectories through developmental programming mechanisms and highlight the importance of adequate maternal protein intake during pregnancy for proper tooth development.

In conclusion, our finding emphasizes the temporally specific effects of GPR on autophagy during tooth germ development. The data suggest that GPR impairs autophagic vesicle maturation and degradation, potentially hindering recycling of cellular components, essential for proper cell proliferation and differentiation. This disruption in autophagic flux may contribute to adverse tooth outcomes. Our results indicate the critical role of proper nutrient availability during gestation in regulating developmental processes of the tooth germ, possibly leading to long-term consequences for dental health.

## Supplementary Information

Below is the link to the electronic supplementary material.

Supplementary file1 (PDF 242531 KB)

## Data Availability

The datasets generated and/or analyzed during the current study are available from the corresponding author on reasonable request.
