# Supplementary material for: Effects of gestational protein restriction on autophagy dynamics during odontogenesis
Source: Cell Tissue Res. 2026 Jun 19;404(3):23. doi: 10.1007/s00441-026-04079-0 (PMC13282235; doi:10.1007/s00441-026-04079-0)

Negative controls of immunohistochemistry

Effects of Gestational Protein Restriction on Autophagy Dynamics during Odontogenesis

Authors: Calsa B, Gontijo JAR, Santamaria-JR M, Boer PA

File content

- Image 1: Rabbit control – peroxidase.
- Image 2: Mouse control – peroxidase.
- Image 3: Mouse control – fluorescence.
- Image 4: - Rabbit control – fluorescence.

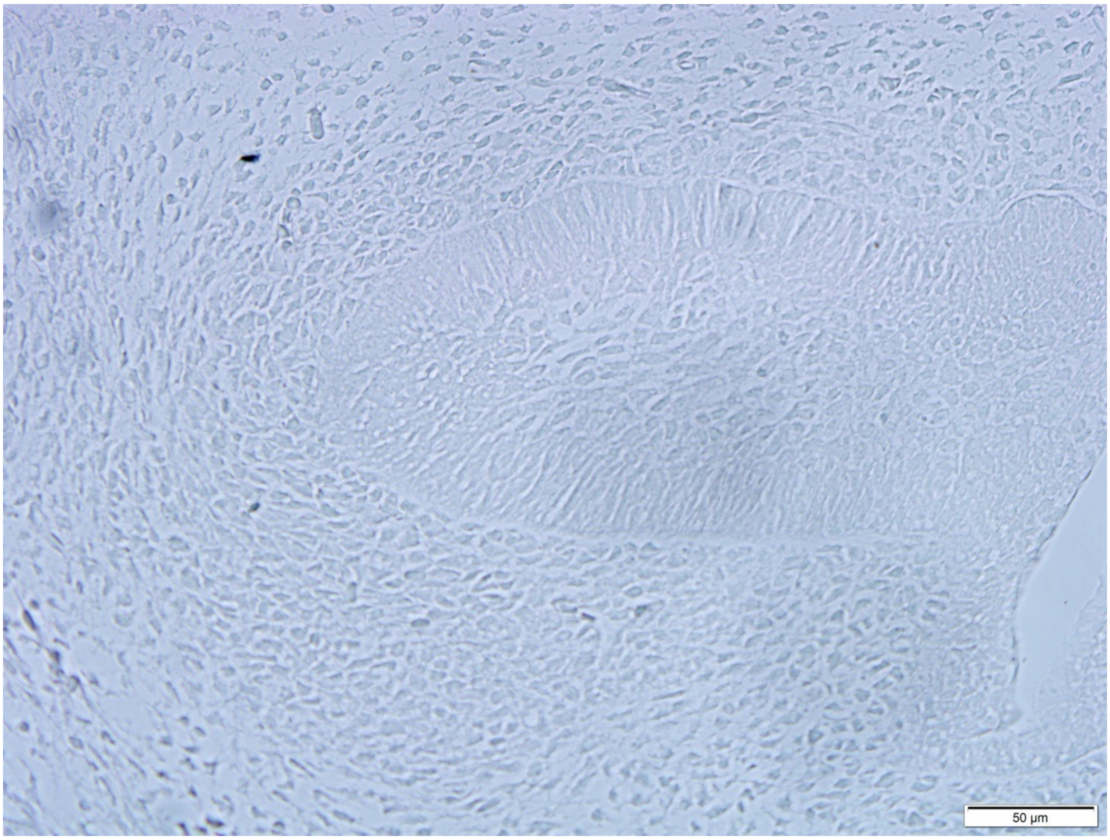

Rabbit negative control. The primary antibody was omitted, istead, the tissue was treated with 3% BSA.

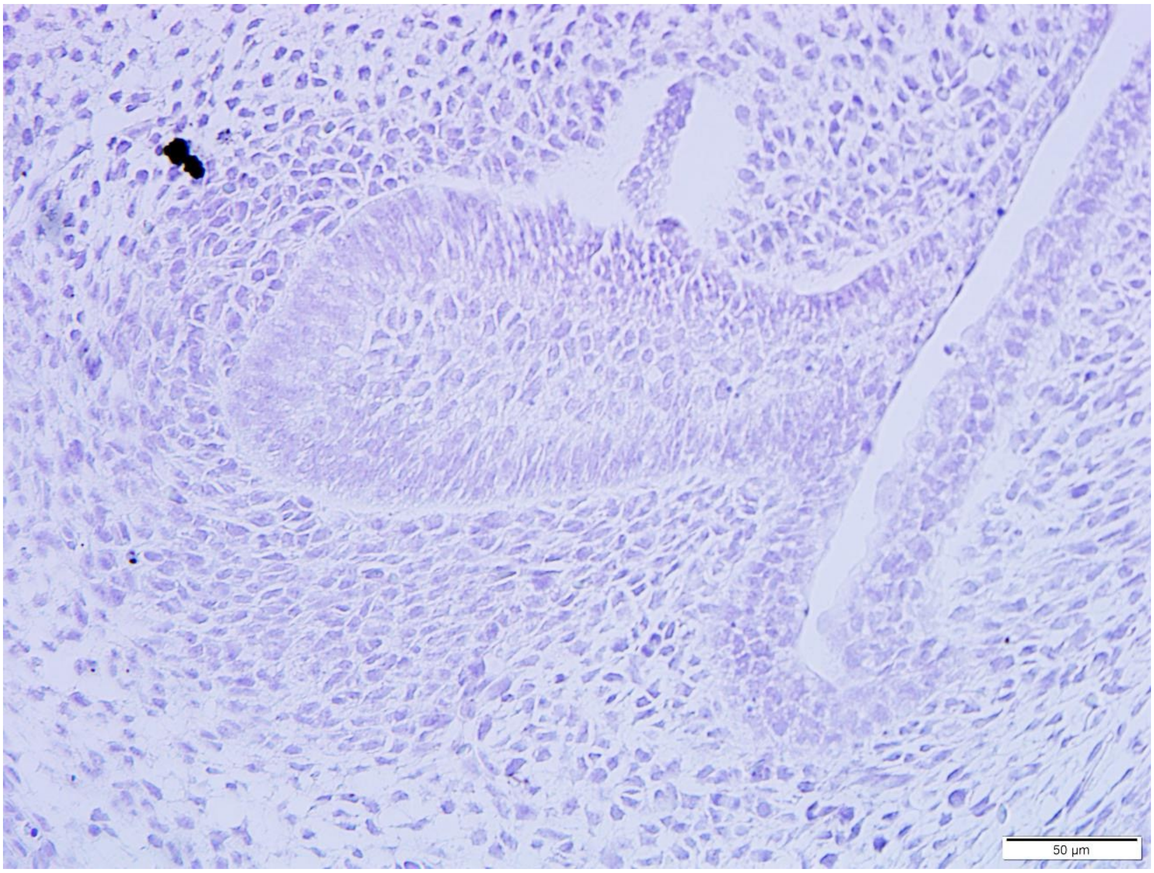

Mouse negative control. The primary antibody was omitted, istead, the tissue was treated with 3% BSA. The section was counterstained with hematoxylin.

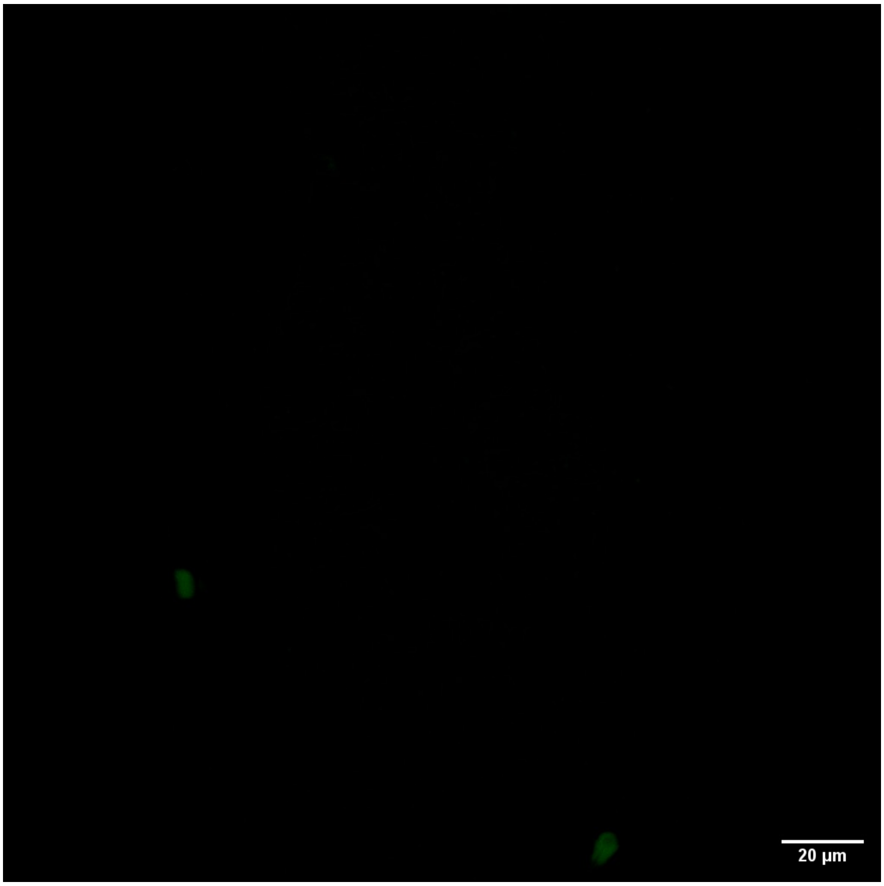

Mouse negative control. The primary antibody was omitted, istead, the tissue was treated with 5% non-immune goat serum.

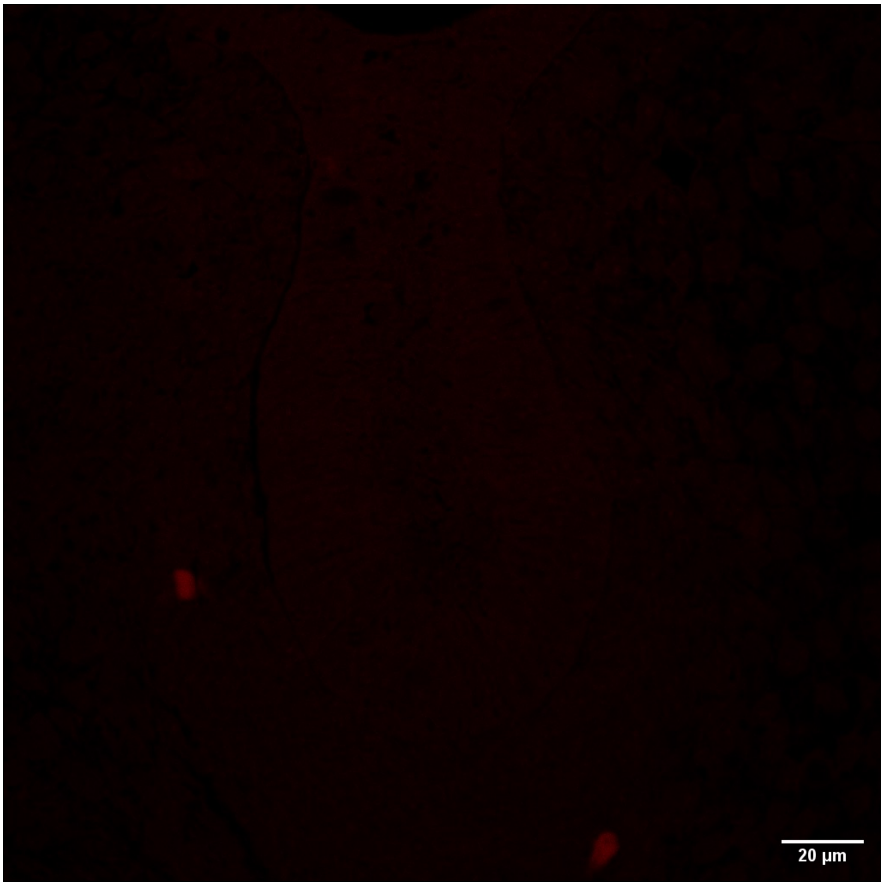

Rabbit negative control. The primary antibody was omitted, istead, the tissue was treated with 5% non-immune goat serum.

Supplementary file 2

Representative panels for autophagy assessment 14GD

Effects of Gestational Protein Restriction on Autophagy Dynamics during Odontogenesis

Authors: Calsa B, Gontijo JAR, Santamaria-JR M, Boer PA

File content

- Panel 1: Whole tooth germ at 14GD.
- Panel 2: Dental epithelium at 14GD.
- Panel 3: Dental mesenchyme at 14GD – separated channels.
- Panel 4: Dental mesenchyme at 14GD – merged channels.

Panel 1

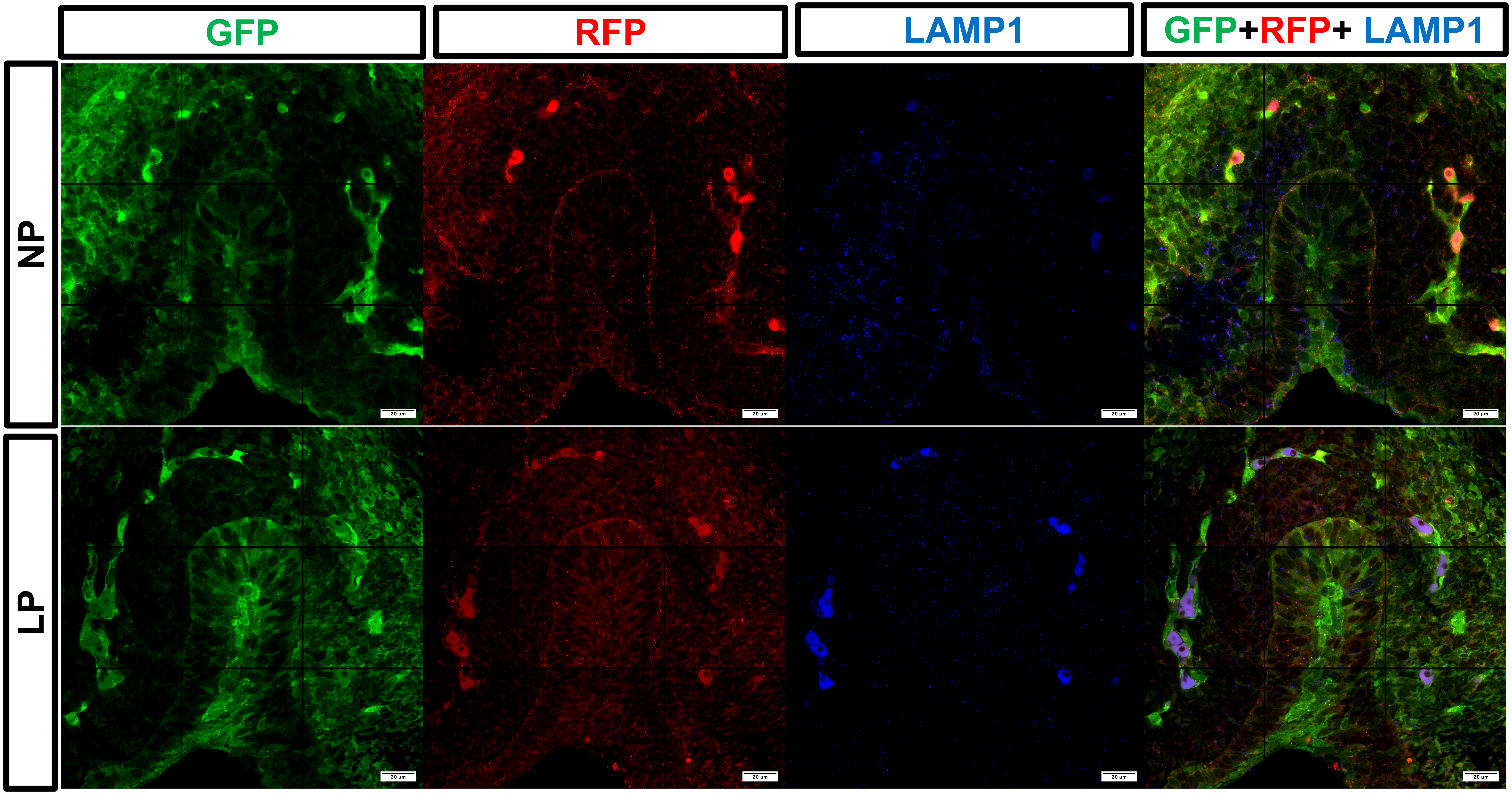

Panel 2

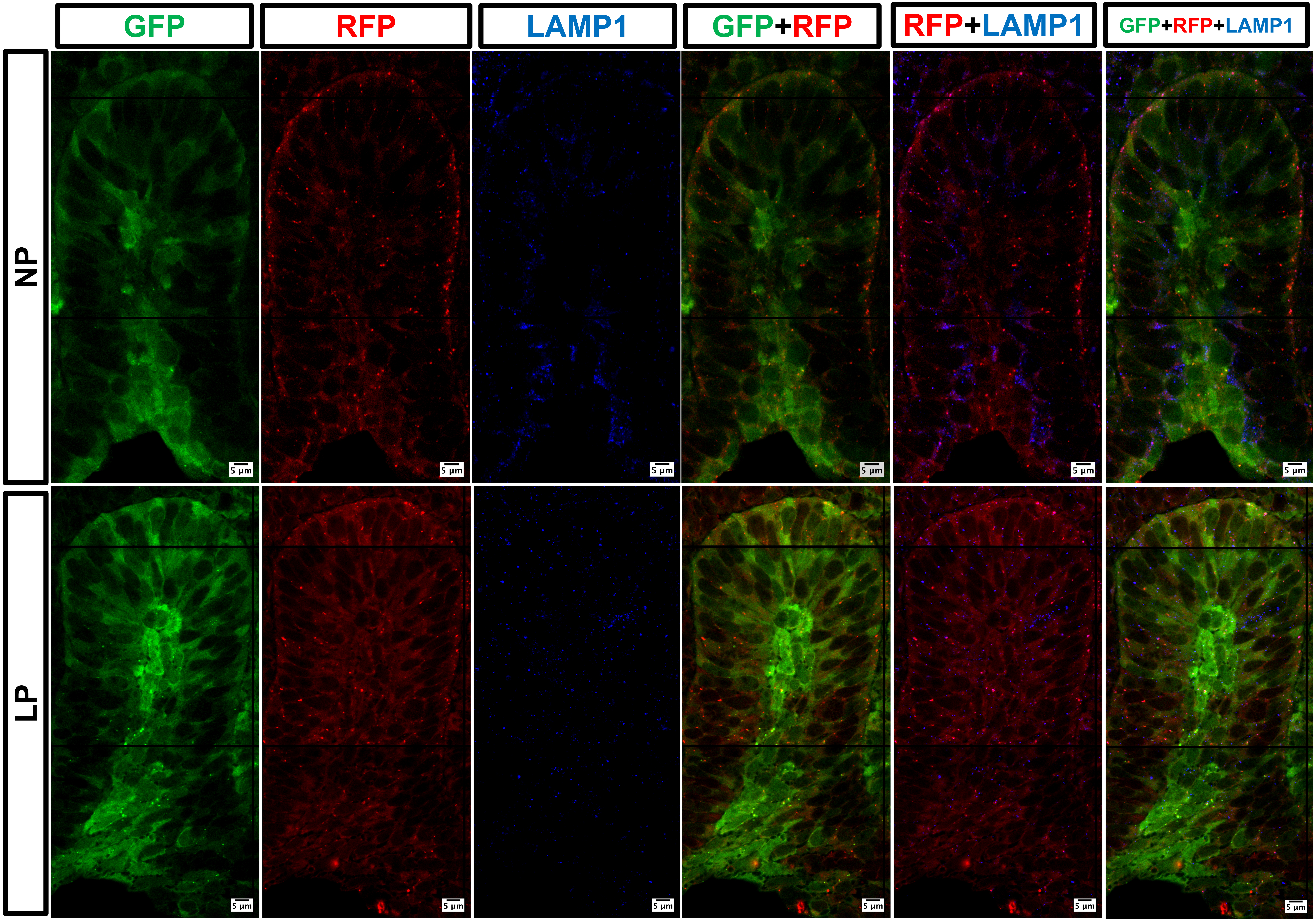

Panel 3

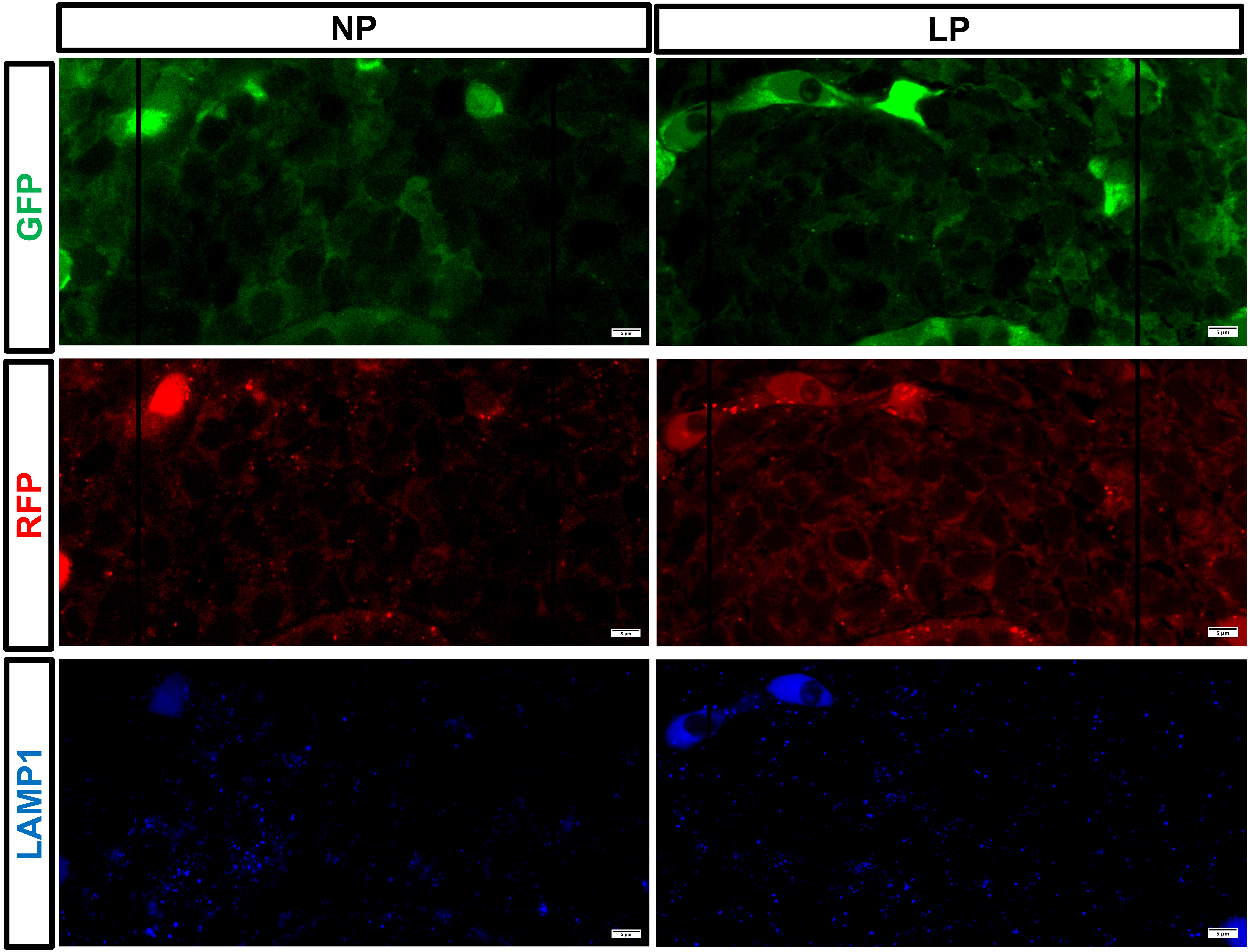

Panel 4

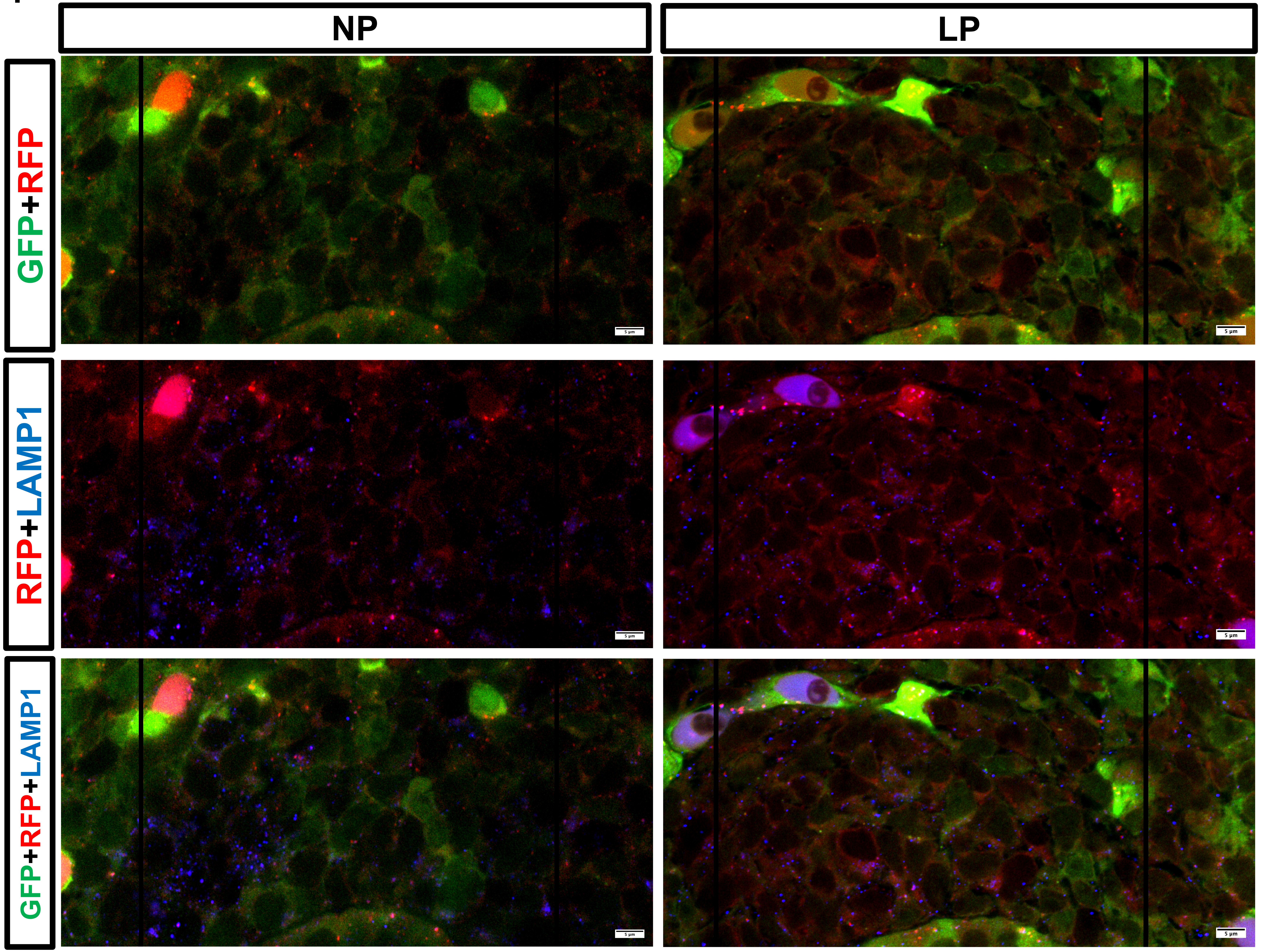

Supplementary file 3

Representative panels for autophagy assessment 16GD

Effects of Gestational Protein Restriction on Autophagy Dynamics during Odontogenesis

Authors: Calsa B, Gontijo JAR, Santamaria-JR M, Boer PA

File content

- Panel 1: Whole tooth germ at 16GD.
- Panel 2: Dental papilla at 16GD – separated channels.
- Panel 3: Dental papilla at 16GD – merged channels.
- Panel 4: Enamel organ at 16GD – separated channels.
- Panel 5: Enamel organ at 16GD – merged channels.

Panel 1

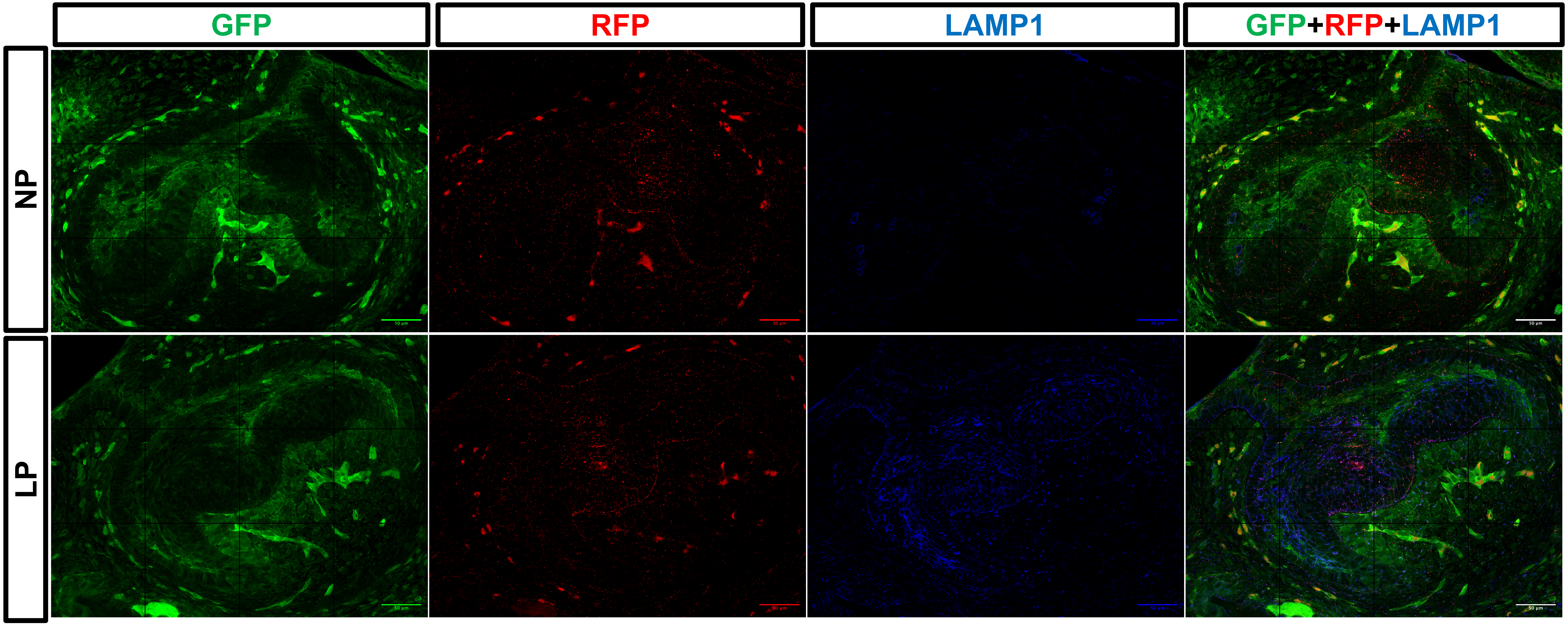

Panel 2

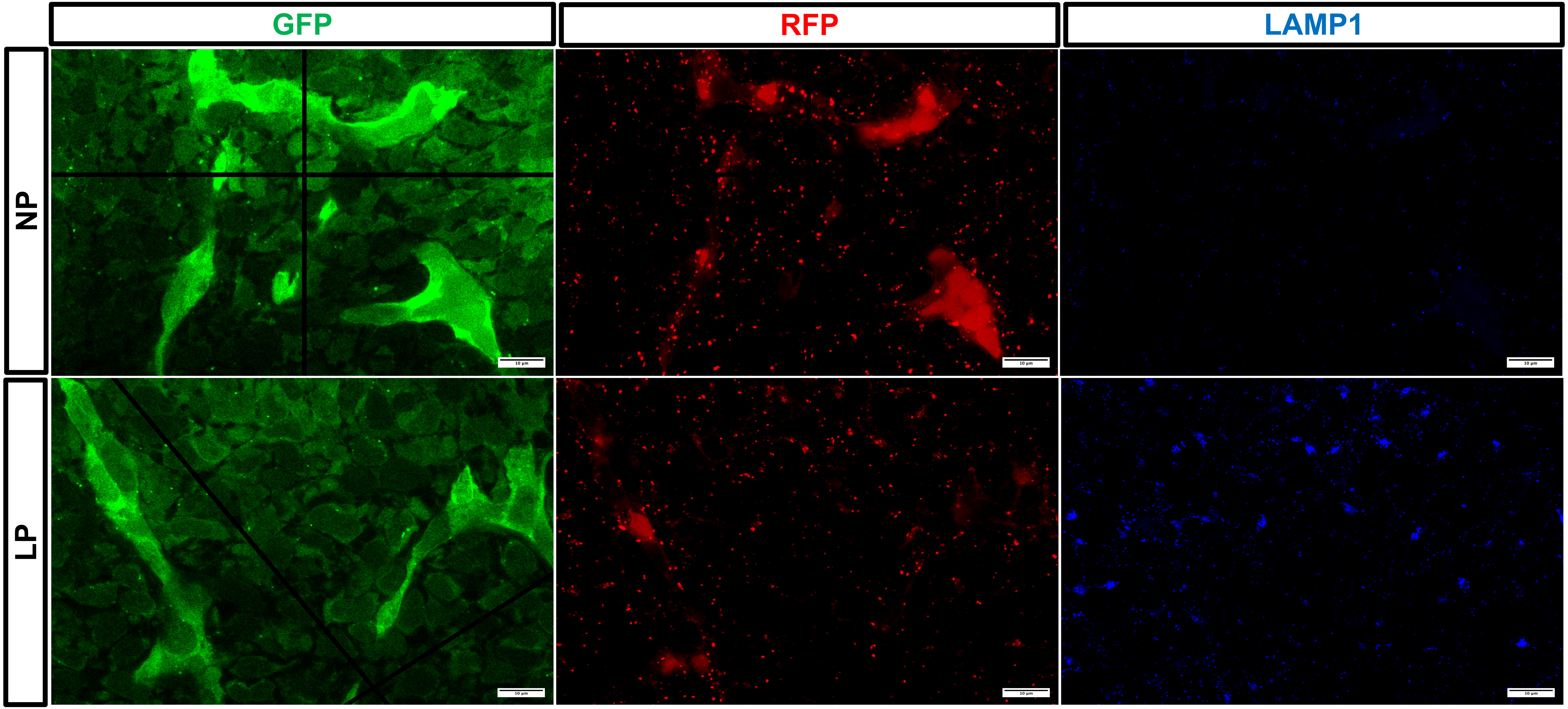

Panel 3

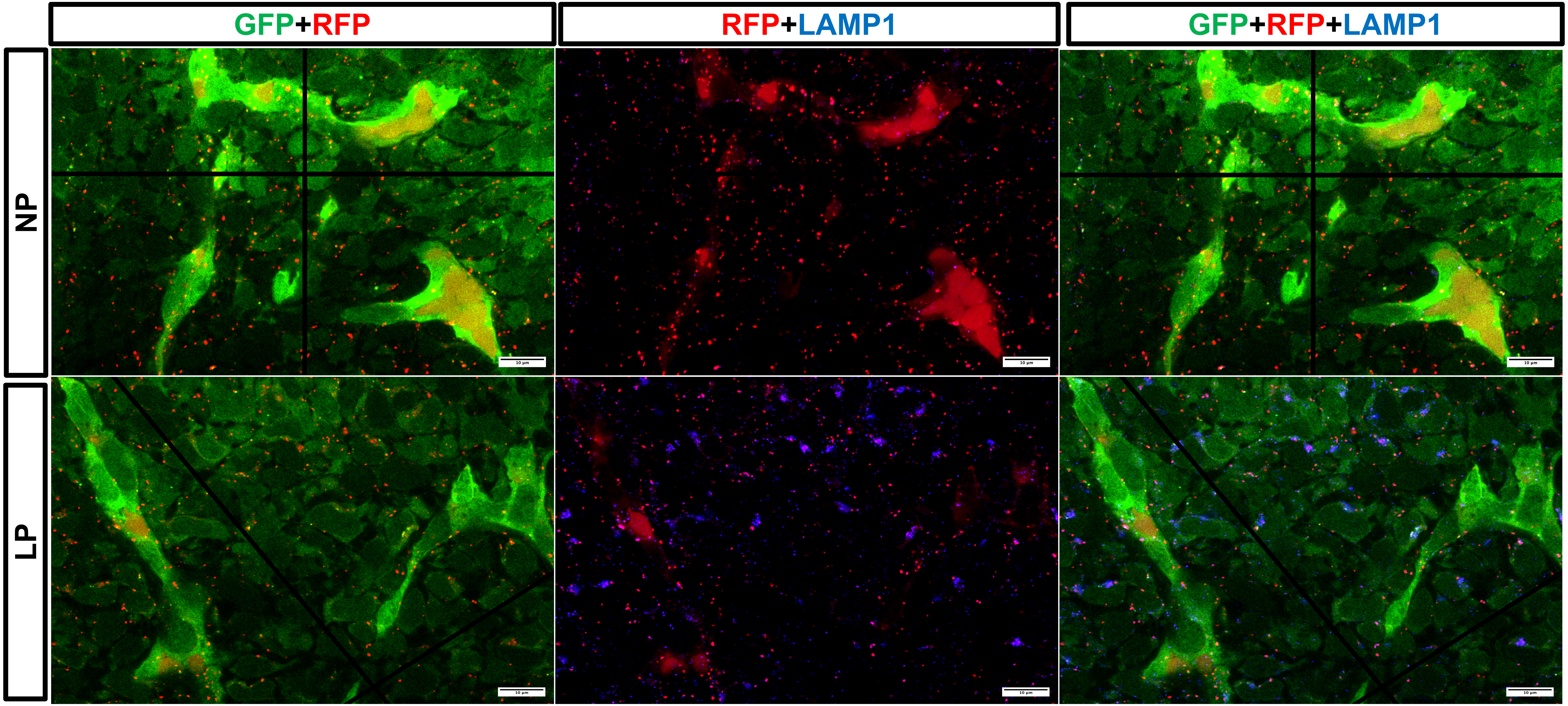

Panel 4

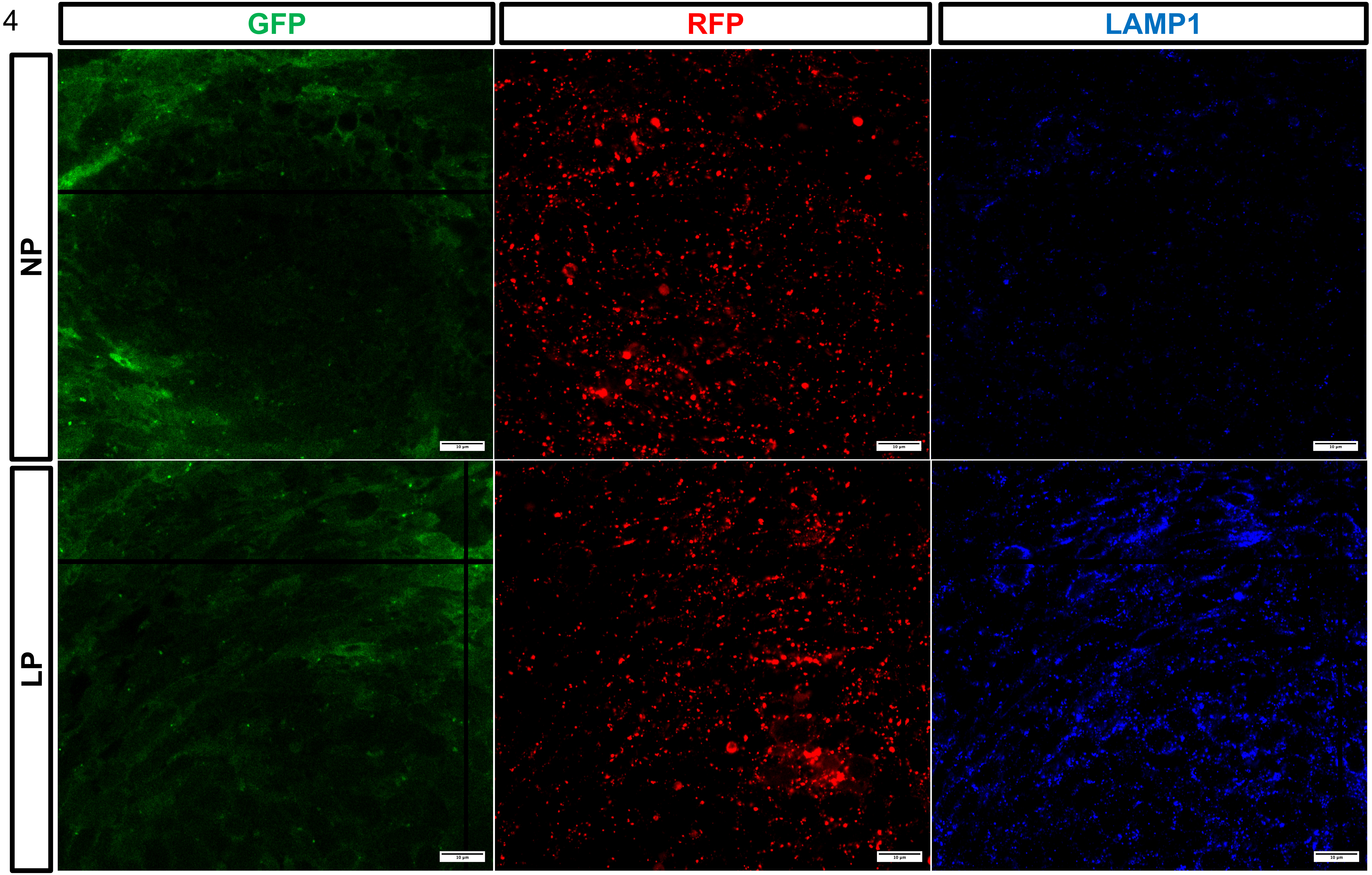

Panel 5

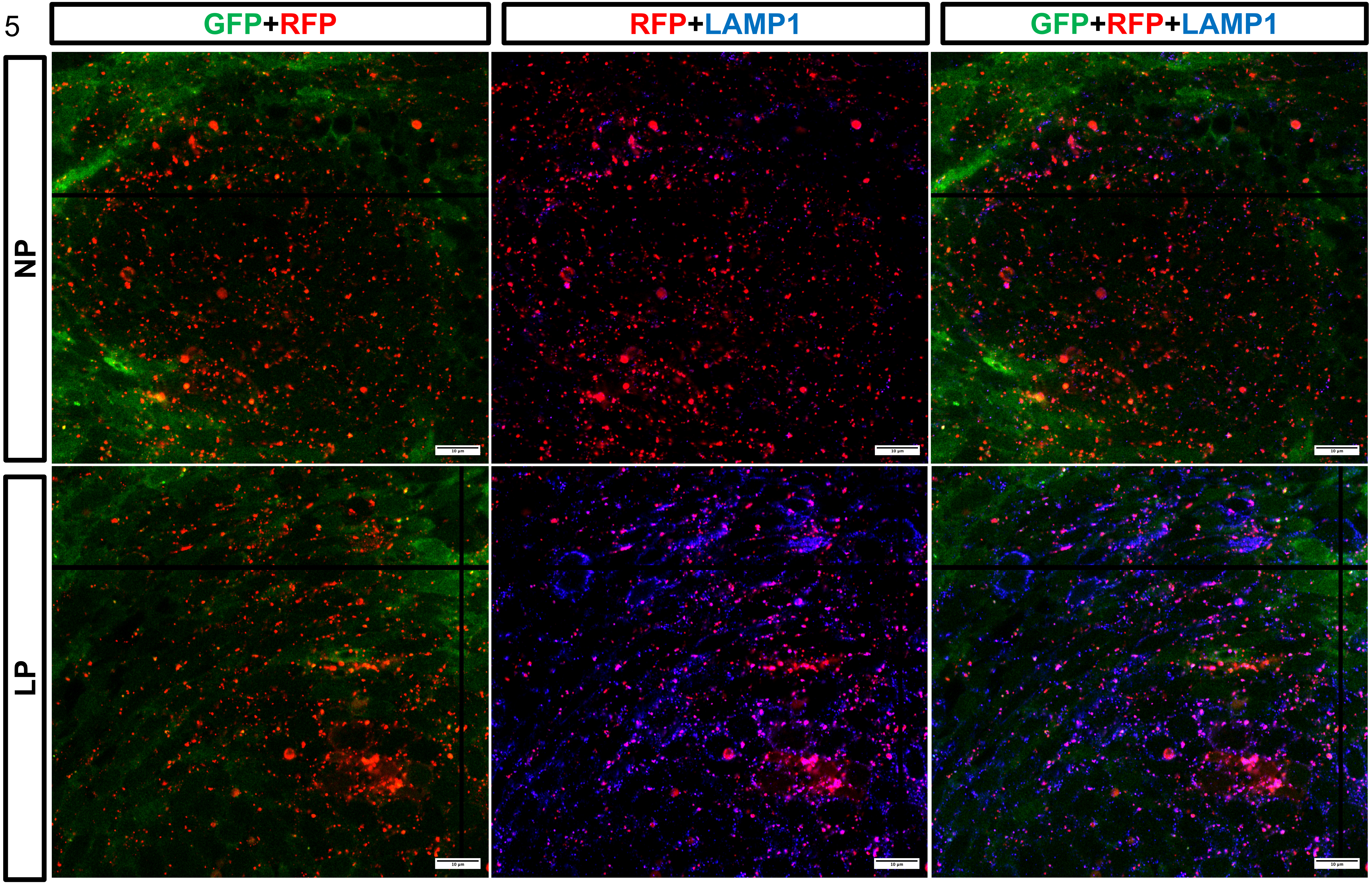

Representative panels for autophagy assessment 18GD

Effects of Gestational Protein Restriction on Autophagy Dynamics during Odontogenesis

Authors: Calsa B, Gontijo JAR, Santamaria-JR M, Boer PA

File content

- Panel 1: Whole tooth germ at 18GD.
- • Panel 2: Dental papilla at 18GD – separated channels.
- • Panel 3: Dental papilla at 18GD – merged channels.
- • Panel 4: Inner epithelium at 18GD – separated channels.
- • Panel 5: Inner epithelium at 18GD – merged channels.

Panel 1

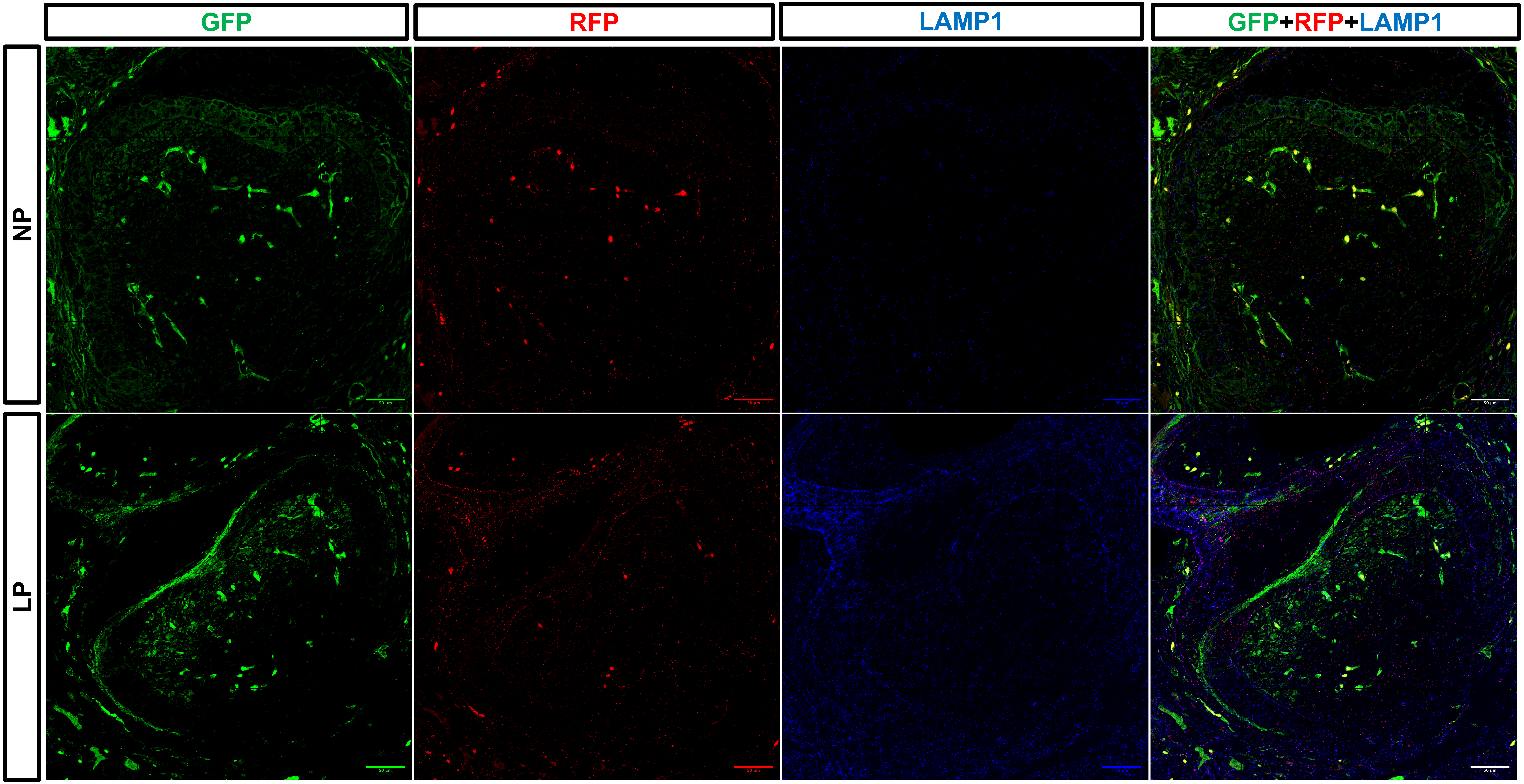

Panel 2

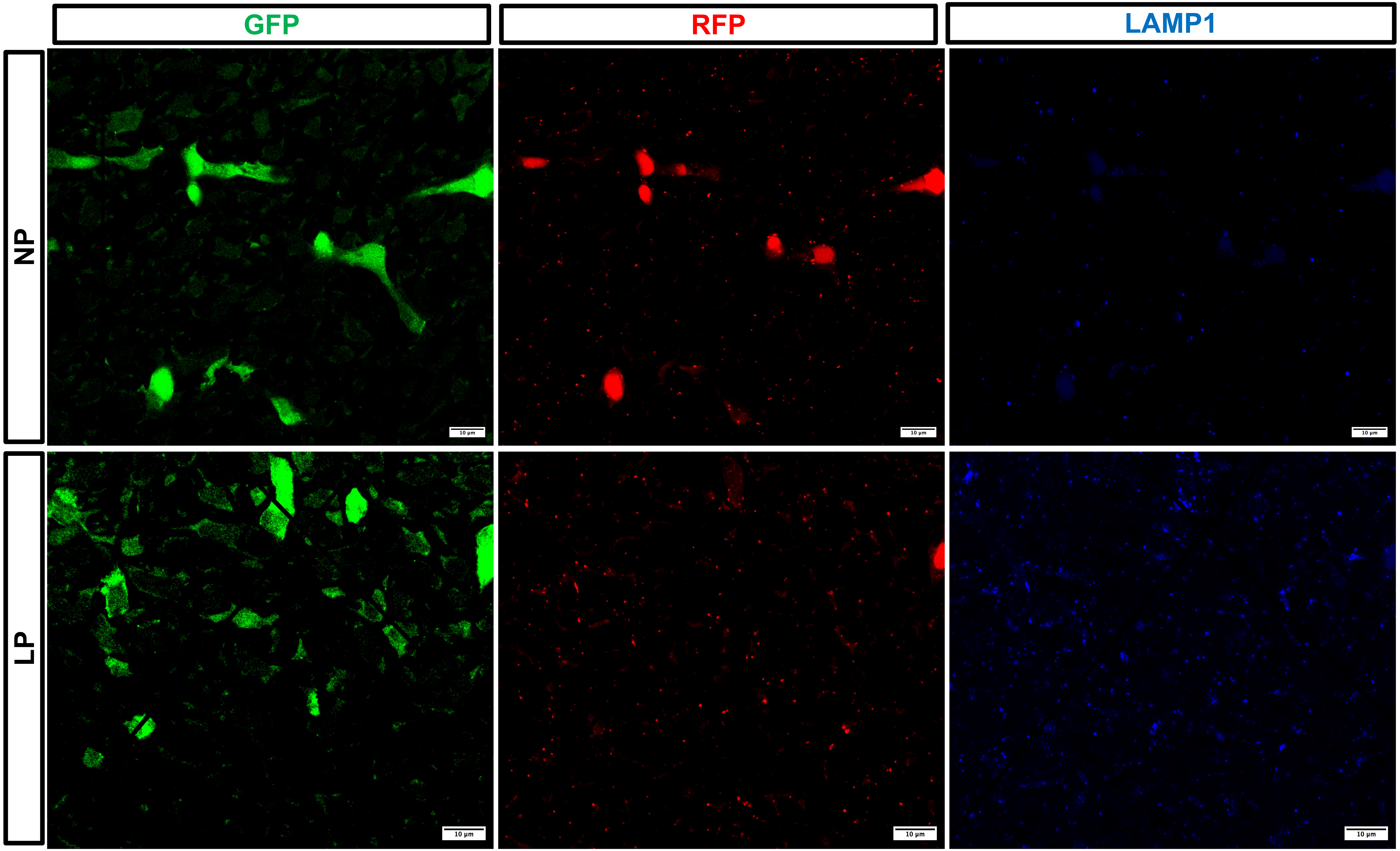

Panel 3

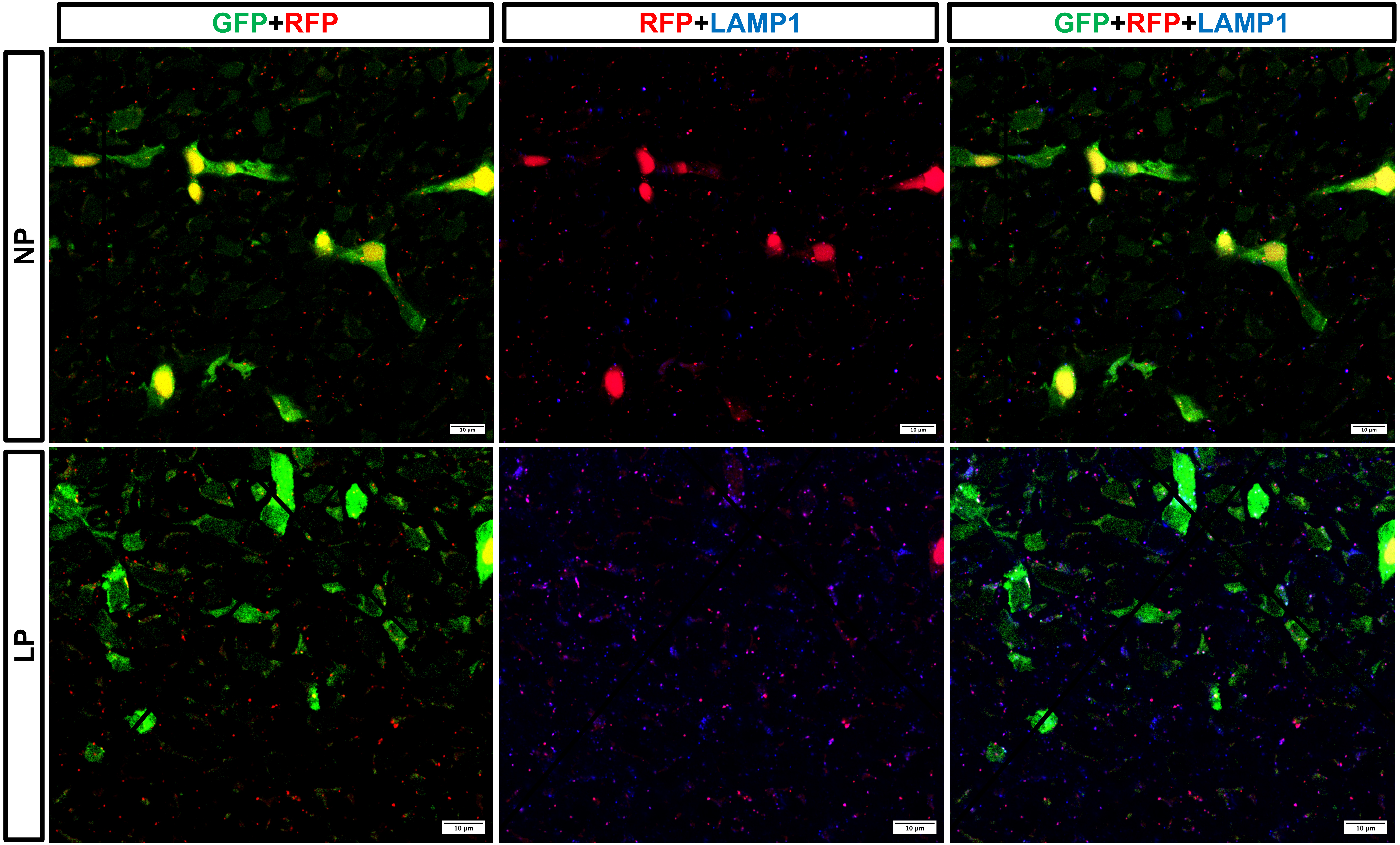

Panel 4

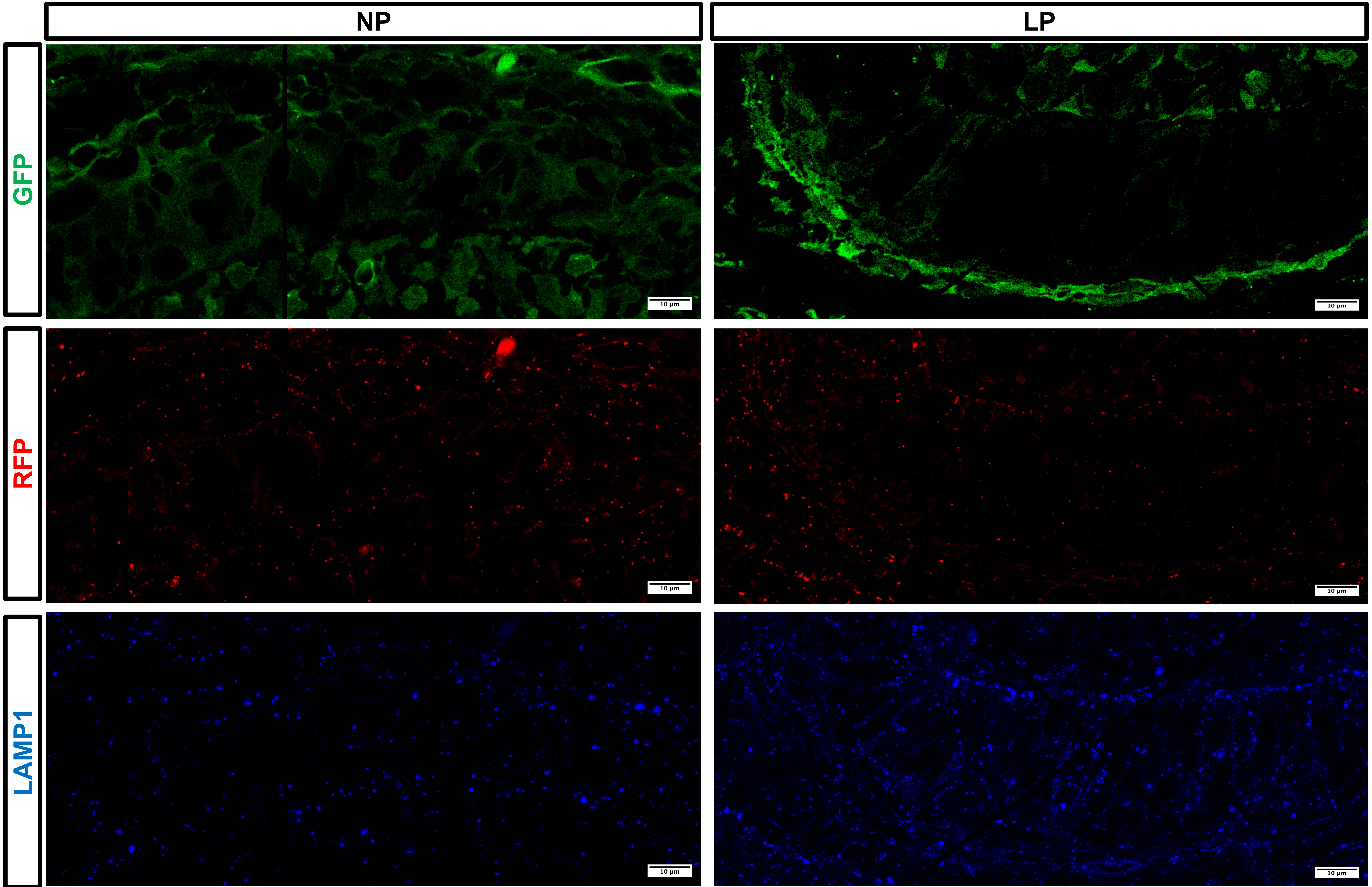

Panel 5

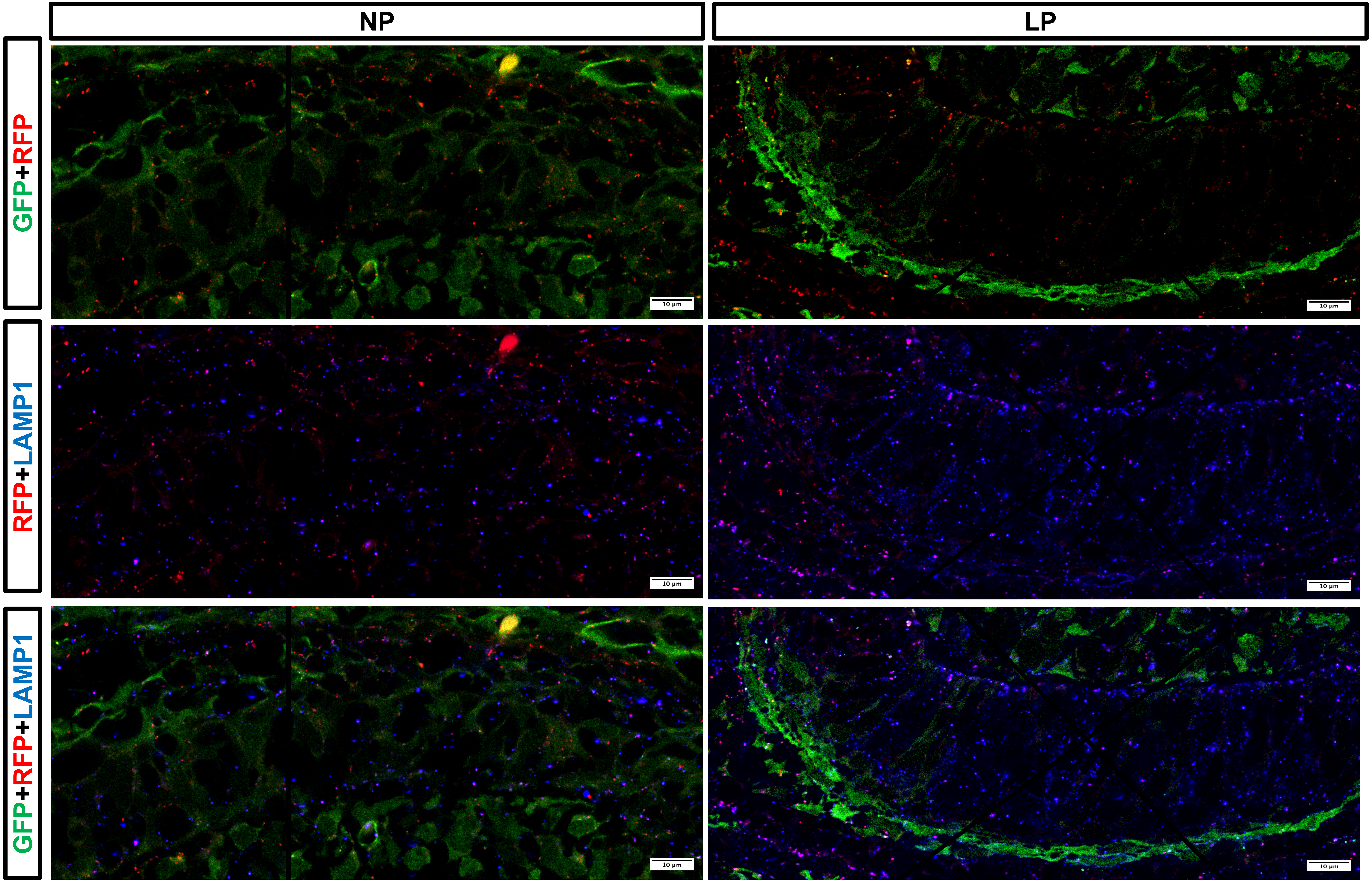

Supplement: Supplementary file 1 — Supplementary file1 (PDF 242531 KB) [file 441_2026_4079_MOESM1_ESM.pdf]
